# Genomic and functional insights into commensal streptococci with anti-pneumococcal activity

**DOI:** 10.1186/s12864-025-11756-x

**Published:** 2025-07-01

**Authors:** Sara Handem, Bárbara Ferreira, Carina Valente, Raquel Sá-Leão

**Affiliations:** 1 Laboratory of Molecular Microbiology of Human Pathogens, Instituto de Tecnologia Química e Biológica António Xavier, Universidade Nova de Lisboa (ITQB NOVA), Avenida da República, Oeiras, 2780-157 Portugal; 2 Polytechnic University of Castelo Branco, Castelo Branco, Portugal

**Keywords:** *Streptococcus mitis*, *Streptococcus oralis*, *Streptococcus pneumoniae*, Commensal, Colonization, Comparative genomics, Capsule, Virulence, Surface protein, Cheater

## Abstract

**Background:**

*Streptococcus pneumoniae* (pneumococcus), *S. mitis*, and *S. oralis* are closely related bacteria that colonize the human upper respiratory tract. While pneumococcus is a leading cause of global morbidity and mortality, *S. mitis* and *S. oralis* are generally considered commensals, rarely causing disease in immunocompetent hosts. Here, we characterized the genomes of seven commensal streptococcal strains (*S. oralis* A22 and *S. mitis* B22–G22) identified as potential biotherapeutics due to their bacteriocin-mediated antipneumococcal activity.

**Results:**

Comparative genomic analyses revealed key differences between these commensals and pneumococci. Commensal strains encode diverse adhesin-like proteins, absent in pneumococci, and lack key virulence factors such as pilus islets and pneumococcal surface proteins. They also possess extensive restriction-modification and type II toxin-antitoxin systems, alongside novel prophages, suggesting roles in genetic stability and phage defense. Metabolic adaptations in commensals indicate a “cheater” strategy, relying on extracellular metabolites from other microorganisms, particularly in the nutrient-scarce nasopharynx. Additionally, commensal strains exhibit distinct teichoic acid compositions, with galactose-rich lipoteichoic acids potentially enhancing niche adaptation. Capsular diversity was also observed, with some strains encoding unique polysaccharides. These findings highlight genomic features that likely enhance commensal colonization and survival in the upper respiratory tract, while distinguishing them from pneumococci.

**Conclusions:**

This study highlights the genomic characteristics of the seven commensal streptococcal strains with broad anti-pneumococcal activity recently described and provides insights into species-specific traits that could inform targeted strategies for pneumococcal control.

**Supplementary Information:**

The online version contains supplementary material available at 10.1186/s12864-025-11756-x.

## Background

Streptococci of the Mitis group (SMG) inhabit the human upper respiratory tract (URT) and exhibit diverse pathogenic traits. *S. pneumoniae* is a common URT colonizer but also a leading cause of morbidity and mortality, particularly in young children, the elderly, and the immunocompromised [1, 2]. In contrast, *S. mitis* and *S. oralis* are typically commensals, rarely causing disease except in high-risk individuals [3–6].

Numerous studies have investigated pneumococcal virulence factors and the transition from colonization to a pathogenic state (reviewed in [7, 8]). Of interest, key virulence factors, such as the polysaccharide capsule, immunoglobulin A1 protease, and lysins, may also be found in commensal streptococci [9, 10]. This suggests that pathogenicity depends on attributes beyond the mere presence of virulence factors and that virulence factors may have additional, non-virulence related, functions [9, 10]. While pneumococcal colonization is often asymptomatic and transient - lasting from days to months - it involves a dynamic cycle of strain acquisition, immune response activation, and eventual clearance [11–14]. In contrast, *S. mitis* and *S. oralis* appear to colonize the host for longer periods, though their relative proportions fluctuate over time [15–17].

Recently, there has been a noticeable increase in publicly available streptococcal genomes. However, while extensive research has been conducted on *S. pneumoniae*, *S. mitis* and *S. oralis* remain understudied [10, 18–24]. Filling this knowledge gap is crucial to better understand differences between closely related commensal and pathogenic species, which could inform targeted strategies to control *S. pneumoniae*.

This study comprehensively characterizes the genomes of seven commensal streptococcal strains - one *S. oralis* and six *S. mitis* - previously identified as potential candidates to prevent pneumococcal colonization due to their broad anti-pneumococcal activity [25]. These strains encode an extensive repertoire of bacteriocin-related loci, which likely contribute to their ability to compete in the nasopharyngeal environment. Importantly, several of these bacteriocin loci were implicated in the observed anti-pneumococcal activity, reinforcing the hypothesis that these antimicrobial peptides play a crucial role in bacterial interactions within polymicrobial communities [25]. In the present study, our in-depth analyses focused on key features including surface-exposed proteins, carbohydrate metabolism, restriction-modification and type II toxin-antitoxin systems, bacteriophages, polysaccharide capsule, teichoic acid biosynthesis, and regulatory two-component systems.

Overall, we identify genomic traits that distinguish commensal *S. mitis* and *S. oralis* from *S. pneumoniae*, highlighting factors that likely contribute to their differing pathogenic potentials.

## Methods

### Study strains, DNA extraction and sequencing

Strains *S. oralis* A22 and *S. mitis* B22 to G22 were recently described [25]. These commensal strains, isolated from the upper respiratory tract of healthy subjects display anti-pneumococcal activity against a diverse collection of *S. pneumoniae* which includes various serotypes and genotypes [25].

DNA was extracted and sequenced using Illumina Miseq and Oxford Nanopore MinION technologies [25]. Gene synteny was evaluated using Mauve Snapshot 2015-02-25 [26]. Circular genomes were visualized using Proksee (https://proksee.ca/) [27].

###  In silico genomic characterization

Homologues of pneumococcal genes encoding surface proteins, carbohydrate metabolism, restriction-modification (RM) systems, type II toxin-antitoxin (TA) systems, teichoic acid biosynthetic proteins, and two-component systems (TCS), were identified using BLAST (Table S1 and Method S1) [28]. Additional homologues were searched as follows: surface proteins via CW-PRED [29] and PSORTb v3.0.3 [30] for LPXTG proteins, and PRED-LIPO [31] for lipoproteins; choline-binding proteins (Cbp) via conserved domains (Table S2) and phylogenetic analysis (Method S1); carbohydrate metabolism, TA systems, and TCS via conserved functional domains (Table S2); RM systems were identified using Restriction-ModificationFinder-1.1. Capsule-like proteins were searched by inspecting the genes located between *dexB* and *aliA*. All predicted proteins were validated by BLASTp against NCBI’s non-redundant database (Method S2). Phage regions were identified using PHASTER [32], and annotated using Prokka v1.13.3 [33], Glimmer v0.2 [34], MetaGeneAnnotator v1.0.0 [35], and tRNAscan v0.4 through the Center for Phage Technology Web Galaxy [36], with BLASTp confirmation against NCBI non-redundant and UniProt databases. Phage genomic termini and packing mode were predicted using PhageTerm v1.0.11 [37] and by phylogenetic analysis of the large terminase subunit [38]. Phage taxonomy was determined by genome-wide proteomic phylogenetic analysis using ViPTree v1.9.1 [39]. Transposons were identified by searching genes containing mobile element superfamily domains (Transpos_X superfamily, cl00213 DNA_BRE_C and cl02788 Ser_Recomb), and by further inspecting the adjacent genomic areas.

### Transmission electron microscopy

Strains were grown at 37 °C in Todd-Hewitt broth supplemented with 0.5% yeast extract (THY) to an OD_600nm_ of 1.0 (early stationary phase). Cells were collected and fixed using lysine acetate [40]. Sections were examined using a FEI Tecnai 120 G2 Biotwin Transmission Electron Microscope (80 kV); images were collected using an Olympus-SIS Veleta CCD camera at the Electron Microscopy Unit of Instituto Gulbenkian de Ciência (Oeiras, Portugal).

### Phage induction with mitomycin C

Strains were grown at 37 °C in 6 ml C + Y until an OD_600nm_ of 0.2, divided, and either left untreated or treated with mitomycin C (0.1 µg/ml or 0.5 µg/ml if lysis was not observed). Growth was monitored for 6 h. Pneumococcal strains R6 (non-lysogenic laboratory strain) [41] and 106-1 (lysogenic) [42] served as controls.

### Data Availability

Reads and assembled annotated complete genomes of strains A22-G22 were deposited in European Nucleotide Archive (ENA) under the BioProject accession number PRJEB75690 (Table S3). Reads and annotated genomes of 13 prophages were deposited in NCBI under BioProject accession number PRJNA784746 (Table S3).

## Results

### Seven novel complete genomes were obtained from commensal streptococci isolated from human nasopharyngeal swabs

We previously identified seven commensal streptococci with inhibitory activity against a vast collection of *S. pneumoniae* strains [25]. These included one of *S. oralis* subsp. *oralis* (strain A22) and six *S. mitis* (named B22 to G22) [25]. Their genomes were sequenced, and their main genomic features are summarized in Table 1. All strains displayed a symmetrical GC-skew profile with coding sequences oriented predominantly with the GC-skew, typical of low-GC Gram-positive species (Fig. S1). Their genomic characteristics, including size, GC content and gene count, aligned with complete *S. mitis* and *S. oralis* genomes in the NCBI database. Few complete genomes exist for these species, with 11 for *S. mitis* (10 at the time of our analysis) and 16 for *S. oralis subsp. oralis*, most from non-nasopharyngeal sites (Table S4). Our study adds seven fully sequenced genomes from nasopharyngeal carriage. Gene synteny was conserved among *S. mitis* strains, except for D22, which showed extensive chromosomal rearrangements. In contrast, *S. oralis* strain A22 exhibited synteny with *S. mitis* only at genome ends (Fig. S2).

**Table 1:** Genomic characteristics of the seven commensal Streptococcal strains

Species	Strain	Size (bp)^a^	% GC	Genes	CDS	tRNA
*S. oralis*	A22	1,956,731^b^	41.3	2,009	1,918	62
*S. mitis*	B22	2,097,606	40.3	2,029	1,926	59
	C22	2,178,839^c^	40.1	2,088	1,987	59
	D22	2,260,119^d^	39.6	2,289	2,178	61
	E22	2,148,157^e^	40.3	2,087	1,981	57
	F22	2,160,848	40.2	2,041	1,939	59
	G22	2,184,976	40.2	2,117	2,010	59

% GC– percentage of guanine and cytosine bases in the genome; CDS– coding sequence

^a^ Single contig except if otherwise noted

^b^ Two contigs of 1,952,063 bp and 4,668 bp (6 genes, 6 CDS)

^c^ Two contigs of 2,141,924 bp and 36,915 bp (62 genes, 62 CDS)

^d^ Two contigs of 2,220,448 bp and 39,671 bp (64 genes, 63 CDS)

^e^ Three contigs of 2,084,724 bp, 37,384 bp (64 genes, 64 CDS) and 15,578 bp (19 genes, 19 CDS)

### Commensal streptococci encode a low number of putative virulence factors and many adhesin-like proteins

**Fig. 1:**
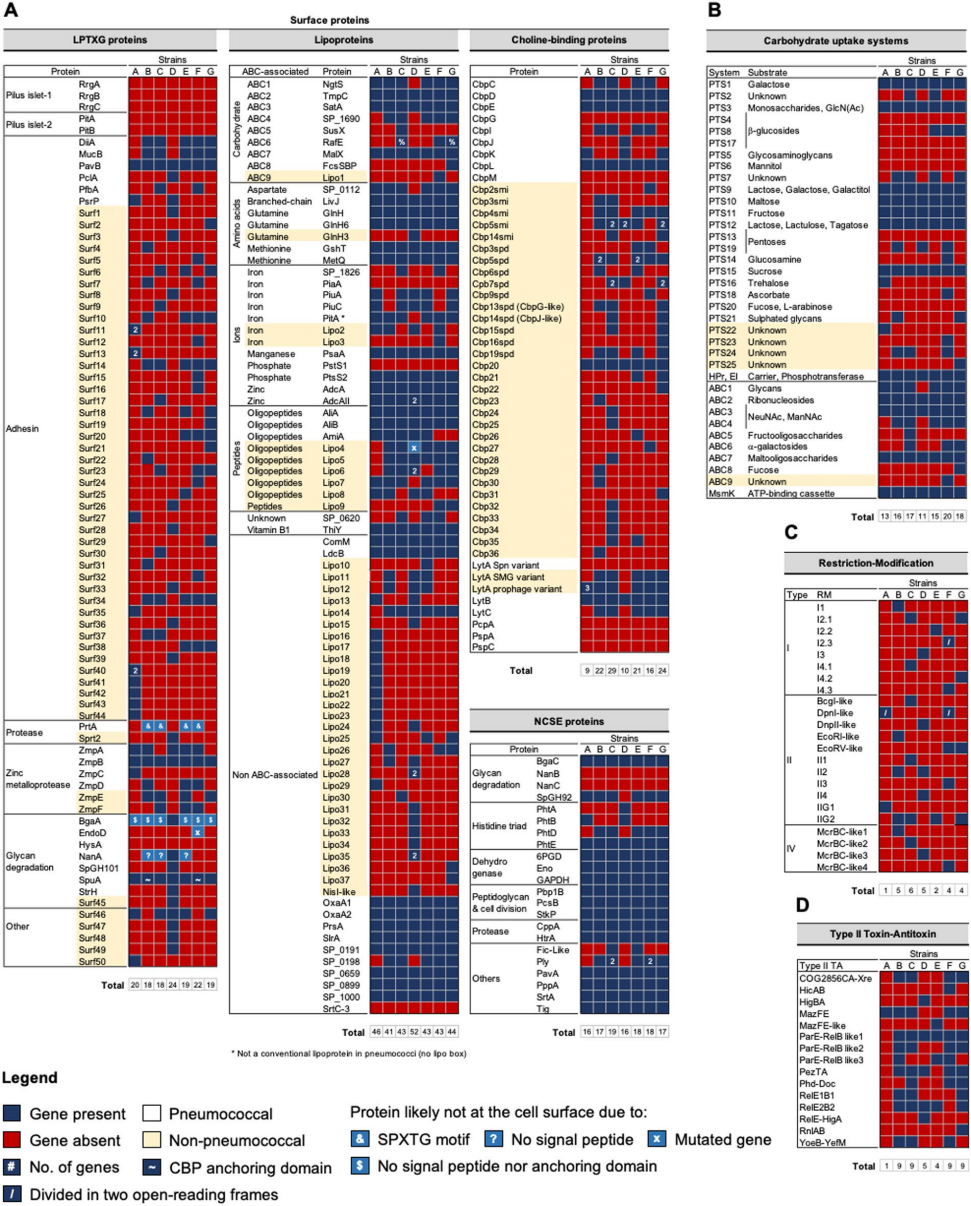
Distribution of surface proteins, carbohydrate-uptake systems, restriction-modification, and type II toxin-antitoxin systems among the commensal streptococcal study strains. Blue squares indicate gene presence; red squares indicate gene absence. Numbers inside blue squares indicate the number of homologue genes for a given protein. Additional marks represent protein features of interest. Yellow indicates additional putative proteins found among the study strains that are absent or rare in pneumococci

We searched for homologues to 99 pneumococcal surface proteins in the genomes of the seven study strains. These included LPXTG-containing proteins, choline-binding proteins, lipoproteins, and non-classical surface-exposed proteins involved in pneumococcal growth, colonization, invasion, and immune evasion (reviewed in [43]). Additionally, putatively exposed proteins were analyzed to infer their structural features. The strains encoded between 89 (S. oralis strain A22) and 106 (S. mitis strain C22) putative surface proteins (Fig. 1A and Table S5A). In general, S. mitis strains encoded slightly more surface proteins than the average described for pneumococci [43, 44]. 

**Fig. 2:**
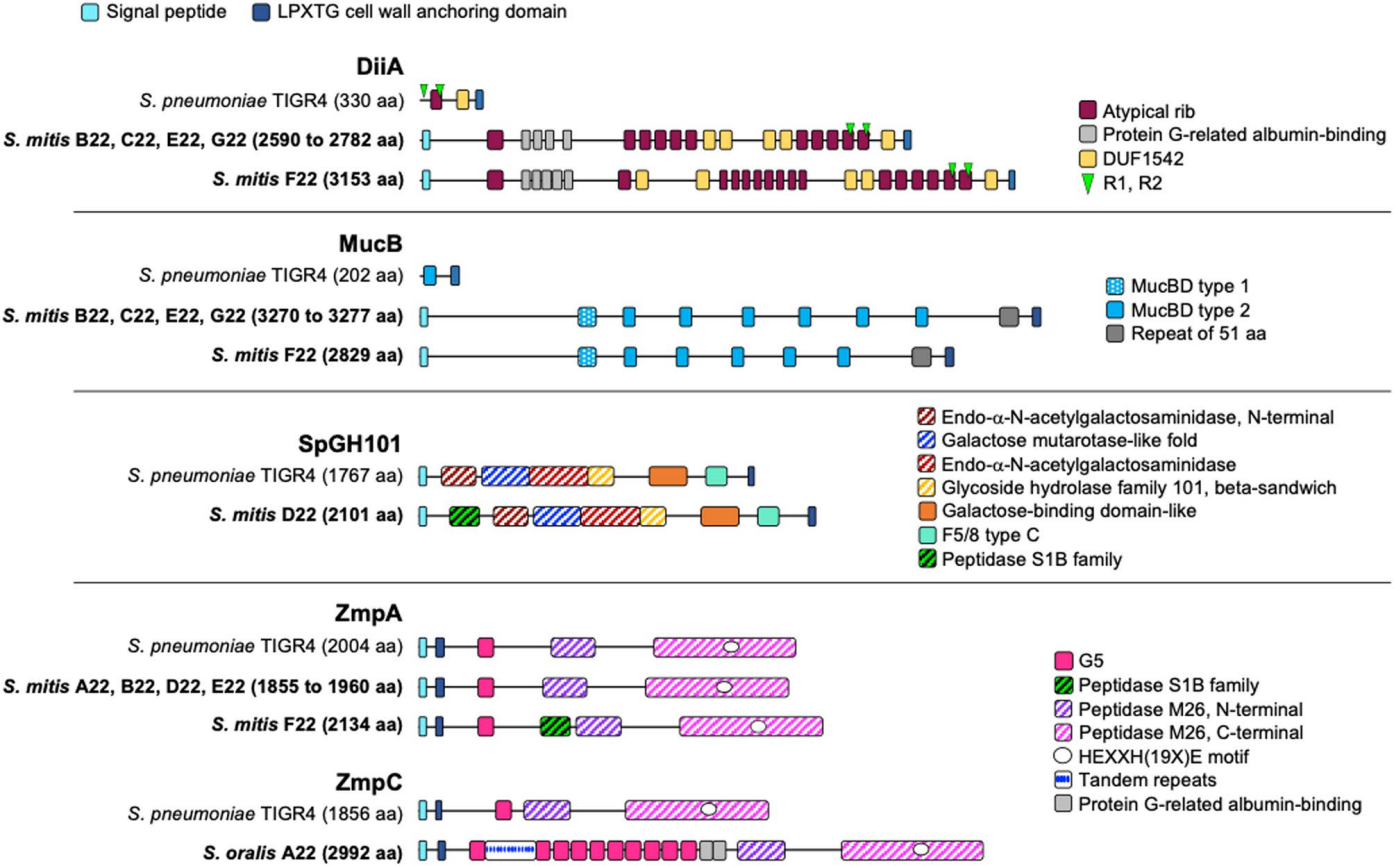
Predicted structural organization of homologues of pneumococcal LPXTG-containing proteins found in commensal streptococcal study strains. Pneumococcal proteins DiiA, MucB, SpGH101, ZmpA, and ZmpC (encoded by SP_1992, SP_0368, SP_1492, SP_1154, and SP_0071, respectively, in *S. pneumoniae* TIGR4) were used for comparison. DiiA-like proteins from commensal study strains were approximately 9-fold longer, had a signal peptide and multiple domain repeats that are absent from the pneumococcal DiiA. MucB-like proteins from commensal study strains were approximately 15-fold longer than the pneumococcal counterpart and displayed more mucin-binding domains (MucBD). SpGH101 and ZmpA from commensal study strains displayed an extra catalytic domain of the peptidase S1B family, and ZmpC had additional repeat regions and multiple G5 domains. DUF– domain of unknown function; F5/8 type C– C-terminal domain of blood coagulation factors V and VIII; G5– domain with conserved glycine residues; GH– glycoside hydrolase; MucBD– mucin-binding domain; R1/R2– aminoterminal motifs enriched in beta-sheets

Among the 99 pneumococcal surface proteins analyzed, homologues for 17, including key virulence factors such as the pilus islets (PI-1 and PI-2), PcpA, PspA, PspC, and HysA, were absent in all seven study strains (Fig. 1A). Forty-five proteins were present in all strains, while the remaining 37 showed variable presence (Fig. 1A). Several homologues showed structural differences from pneumococcal counterparts, potentially influencing function and distinguishing commensal from pathogenic lifestyles. Examples include homologues of LPXTG proteins DiiA, MucB, SpGH101, ZmpA, and ZmpC (Fig. 2). For instance, DiiA-like proteins (found in strains B22, C22, E22, F22, and G22) were approximately nine times longer than pneumococcal DiiA, featuring a signal peptide and multiple domain repeats absent in the latter (Fig. 2). Similarly, MucB-like proteins (strains B22, C22, E22, F22, and G22) were about 15 times longer and contained additional mucin-binding domains (MucBD) (Fig. 2). 

Notably, several additional putative surface proteins were identified in the commensal study strains: of 53 LPXTG proteins, 40 lipoproteins, and 32 choline-binding proteins (Fig. 1A-C, Table S5A). 

**Fig. 3:**
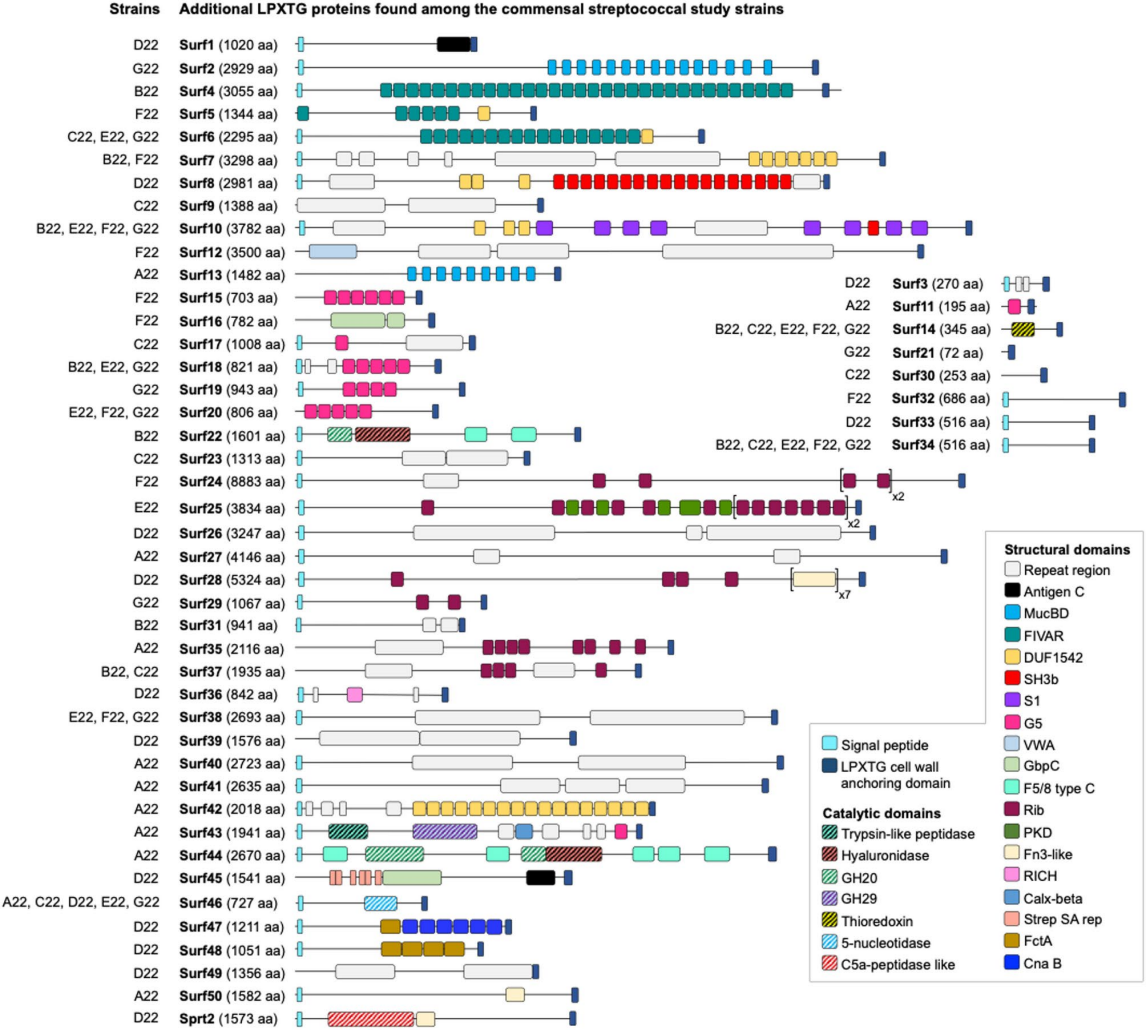
Predicted structural organization of additional (non-pneumococcal) LPXTG-containing proteins found in the commensal streptococcal study strains. Most domains in these proteins are typically found in a variety of cell surface antigens, adhesins, and proteins involved in protein-protein interactions, including of streptococcal species. Calx-beta– cytoplasmic tandem repeat in CalX sodium-calcium exchangers; Cna B– repeated B region domain found in collagen-binding surface protein Cna from *Staphylococcus aureus*; DUF– domain of unknown function; F5/8 type C– C-terminal domain of blood coagulation factors V and VIII; FctA– Spy0128-like isopeptide containing domain; FIVAR– found in various architectures; Fn3-like– fibronectin type 3-like domain; G5– domain with conserved glycine residues; GbpC– glucan-binding protein C; GH– glycoside hydrolase; MucBD– mucin binding domain; PKD– polycystic kidney disease domain; RICH– rich in charged residues; S1– ribosomal protein S1-like RNA-binding domain; SH3b– Src homology-3; Strep SA rep– streptococcal surface antigen repeat; VWA– von Willebrand factor type A

Of the 53 additional LPXTG proteins, here named Surf1 to Surf50, Sprt2, ZmpE, and ZmpF, 40 were strain-specific (Fig. 1A); 19 were large proteins with more than 2,000 amino acids. Most lacked putative catalytic domains but contained signal peptides and tandem repeats of domains commonly found among streptococcal adhesins, suggesting adhesin functions [45, 46] (Fig. 3). BLASTp searches showed that these proteins were predominantly found in *S. mitis* and *S. oralis*, and less frequently in other non-pneumococcal streptococcal species. Noteworthy, all proteins, except Surf21 (a small PsrP-fragment), were rare among pneumococci (Table S6A).

The 40 additional lipoproteins (here named GlnH3, Lipo1 to Lipo37, NisI_like_, and PitA_like_), included 11 associated with ATP-binding cassette (ABC) transporters, while others had unknown functions or metabolic roles (Fig. 1A). BLASTp searches indicated that most were common in *S. mitis*, *S. oralis*, *S. pseudopneumoniae*, *S. infantis*, S. *gordonii*, and *S. sanguinis*, but rare in *S. pneumoniae* (Table S6B).

**Fig. 4:**
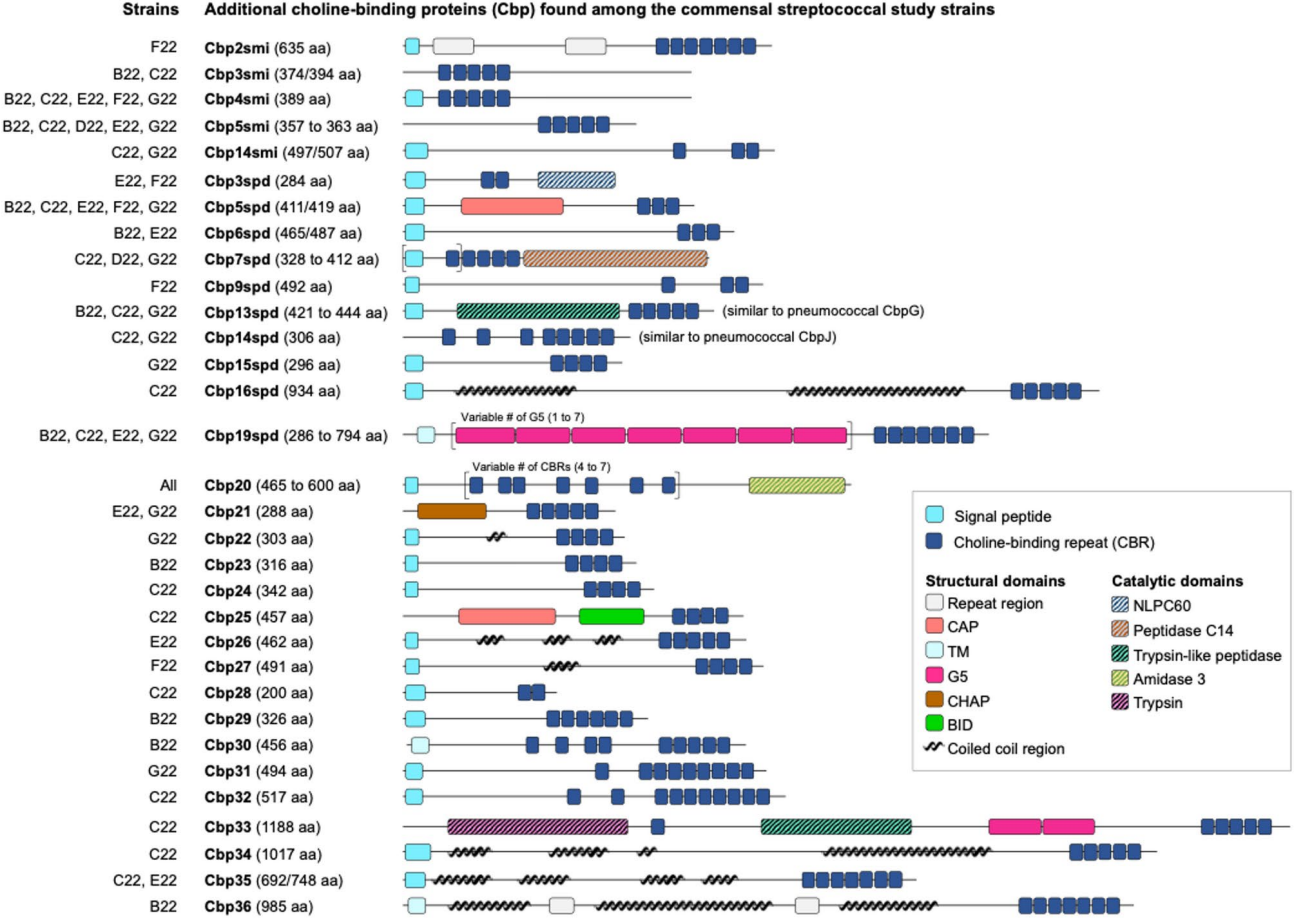
Predicted structural organization of additional (non-pneumococcal) choline-binding protein (Cbp) found in the commensal streptococcal study strains. CbpXsmi have been previously described in *S. mitis* by Denapaite et al. [18]; CbpXspd have been previously described in *S. pseudopneumoniae* by Garriss et al. [47]. Cbp20 to Cbp36 were described and named in the present study. BID– bacterial Ig-like domain; CAP– cysteine-rich secretory proteins, antigen 5, and pathogenesis-related 1 proteins domain; CHAP– cysteine, histidine-dependent aminohydrolase/peptidase; G5– domain with conserved glycine residues; NLPC60– papain-like peptidase domain; TM– transmembrane domain

Of the additional 32 choline-binding proteins, 15 were previously identified in *S. mitis* [18] and *S. pseudopneumoniae* [47], while 17 were novel (named Cbp20 to Cbp36) based on phylogenetic analysis with previously described streptococcal Cbps (Fig. 1A and Fig. S3) [47]. Of those, 13 proteins were strain-specific and one (Cbp20) was encoded by all study strains. Most had signal peptides and choline-binding domains but lacked catalytic domains (Fig. 4), suggesting adhesion roles. Still, three proteins - Cbp20, Cbp21, and Cbp33 - carried catalytic domains (Fig. 4). Cbp20 had a C-terminal amidase domain and Cbp21 (prophage regions in strains E22 and G22) had a N-terminal CHAP domain, suggesting both proteins may function as cell wall degrading enzymes [48]. Cbp33 (strain C22) had a trypsin-like serine protease domain followed by two non-catalytic G5 domains, suggesting a proteolytic role at the cell surface. BLASTp searches indicated that most novel Cbps, although not widespread, were present in *S. mitis*, *S. oralis*, and *S. pseudopneumoniae*, but rare in pneumococci (Table S6C). The exceptions were Cbp21, which was also present in bacteriophages of the *Caudoviricetes* class, and Cbp35 (in strains C22 and E22), which resembled pneumococcal PspA.

In summary, commensal streptococci here described encode fewer *bona fide* virulence factors but more putative adhesin - like proteins than pneumococci. Furthermore, the identification of nearly strain-specific surface proteins - particularly those of the LPXTG and Cbp types - underscores the extensive diversity of cell wall-binding proteins in commensal streptococci, supporting the notion that commensal streptococci are primarily adapted to a colonization lifestyle.

### Commensal streptococci are constrained in the use of carbon sources but may exploit supplementary sources

The genomes of the seven commensal study strains were examined for genes encoding carbohydrate uptake systems and depolymerization proteins for six complex carbon sources. Pneumococci dedicate over 30% of its transport mechanisms to carbohydrate uptake, utilizing 21 phosphoenolpyruvate: sugar phosphotransferase systems (PTS) and eight ABC transporters to import over 30 carbohydrates crucial for colonization and pathogenesis [44, 49]. Of 29 pneumococcal uptake systems, 10 were present in all study strains and mainly associated with common mono- and disaccharides, like galactose, lactose, maltose, and fructose (Fig. 1B and Table S5B). In contrast, six systems associated with the import of b-glucosides, glycosaminoglycans, mannitol, pentoses and fucose, were absent from all strains. Thirteen systems were differentially distributed in the study strains (Fig. 1B and Table S5B). Four additional PTS systems (PTS22 to PTS25), belonging to the mannose-fructose-sorbose family were found in one to three strains (Fig. 1B and Table S5B). BLASTp searches identified the closest homologues of PTS22 (strain A22) in *S. sanguinis*, and of PTS23 (strain F22) and PTS24 (strains B22, C22, and F22) in *S. mitis* and *S. parasanguinis.* The latter was also found in species of *Ligilactobacillus*,* Enterococcus *and* Catenibacterium*, typically found in the gastrointestinal tract. Homologues of PTS25 (strain G22) were found in *Enterococcus*,* Pediococcus* and *Clostridioides* species. One additional ABC transporter (ABC9) was found in strain F22, with the closest homologues being found in *S. mitis*, *S. pseudopneumoniae*, and *S. parasanguinis*. Overall, the commensal study strains encoded fewer carbohydrate uptake systems (11 to 20, average of 16) than pneumococci (20 to 29). 

**Fig. 5:**
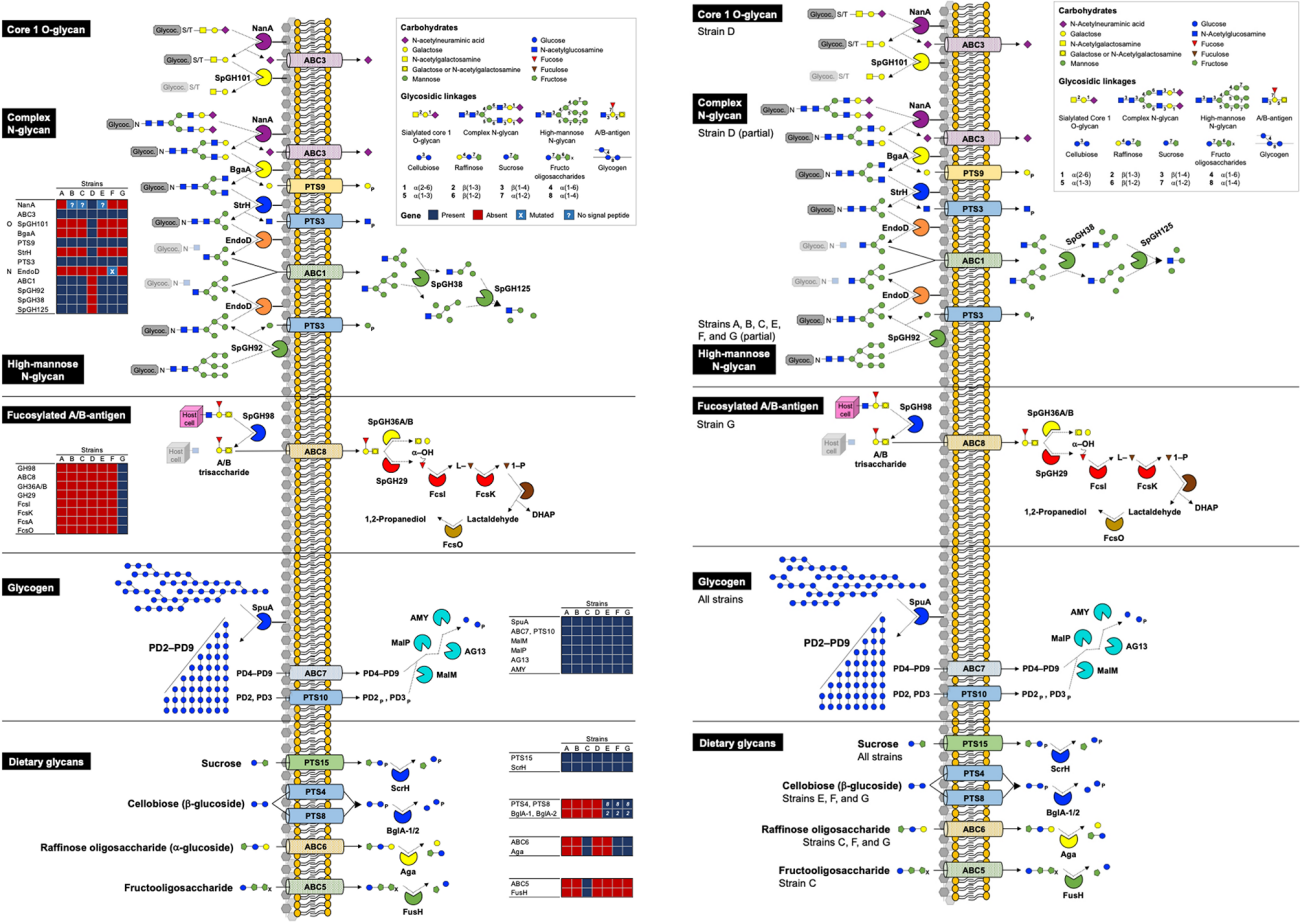
Biosynthetic pathways for the breakdown, uptake, and processing of carbohydrates from O-glycans, N-glycans, fucosylated A/B antigens, glycogen, and dietary glycans. Strain D22 encoded NanA, BgaA, and StrH to obtain sialic acid, galactose, and N-acetylglucosamine, respectively, from core 1 O-glycans and complex N-glycans, and their respective uptake systems ABC3, PTS9, and PTS3. In contrast, the remaining six study strains encoded SpGH92 to obtain mannose from high-mannose N-glycans. All strains lacked EndoD, meaning that they should not be able to release the final mannose core from its glycoconjugate. Nonetheless, all strains except D22 encoded the abovementioned uptake systems for mono-carbohydrates, as well as the transporter ABC1 and intracellular hydrolytic enzymes SpGH38 and SpGH125 to import and further digest the mannose core from N-glycans, suggesting they might be able to use such carbon sources if they are available extracellularly. Strain G22 encoded the proteins to obtain and process fucose from fucosylated A/B antigens (fucose operon type 2). All strains encoded proteins to process glycogen and sucrose (dietary glycan), whereas proteins to process other dietary glycans were differentially found among the study strains (for cellobiose in strains E22, F22, and G22; for raffinose oligosaccharides in strains C22, F22, and G22; and for fructooligosaccharides in strain C22). Figure adapted from Hobbs et al. [50]

Concomitant with the less diversity of carbohydrate uptake systems, and in contrast to pneumococci [50], study strains lacked several carbohydrate-active enzymes necessary for complex carbon source breakdown. Nonetheless, there was variability between strains. For instance, all strains lacked genes for glycosaminoglycans utilization, but all appeared capable of using sucrose (abundant in the diet) and glycogen (abundant in epithelial cells) (Fig. 5). Some strains encoded genes for dietary glycans utilization, such as α-galactosides (strains C22, F22, and G22) and β-glucosides (strains E22, F22, and G22), whereas only one strain (C22) appeared capable of metabolizing fructooligosaccharides or obtaining fucose from fucosylated A/B blood antigens (Fig. 5). Strikingly, all strains except D22 lacked genes for the sequential depolymerization of O- and N-glycans, the dominant glycans in the human airway epithelial cells. Nevertheless, these strains encoded the transporters for importing the intermediate breakdown products (N-acetylneuraminic acid, N-acetylglucosamine, galactose, and mannose), as well as the necessary intracellular hydrolytic enzymes to digest final substrates from both complex and high-mannose N-glycans, suggesting reliance on extracellularly available substrates (Fig. 5). Strain D22, on the other hand, encoded glycan breakdown genes but only to extract their terminal mono-residues, as it lacked genes encoding for proteins to import or further digest the resulting branched products (Fig. 5). 

Taken together, our results suggest that commensal streptococci rely on fewer carbon sources than pneumococci and may adopt a “cheater” strategy to use externally available carbohydrates.

### Commensal streptococci encode a plethora of restriction-modification and type II toxin-antitoxin systems

**Fig. 6:**
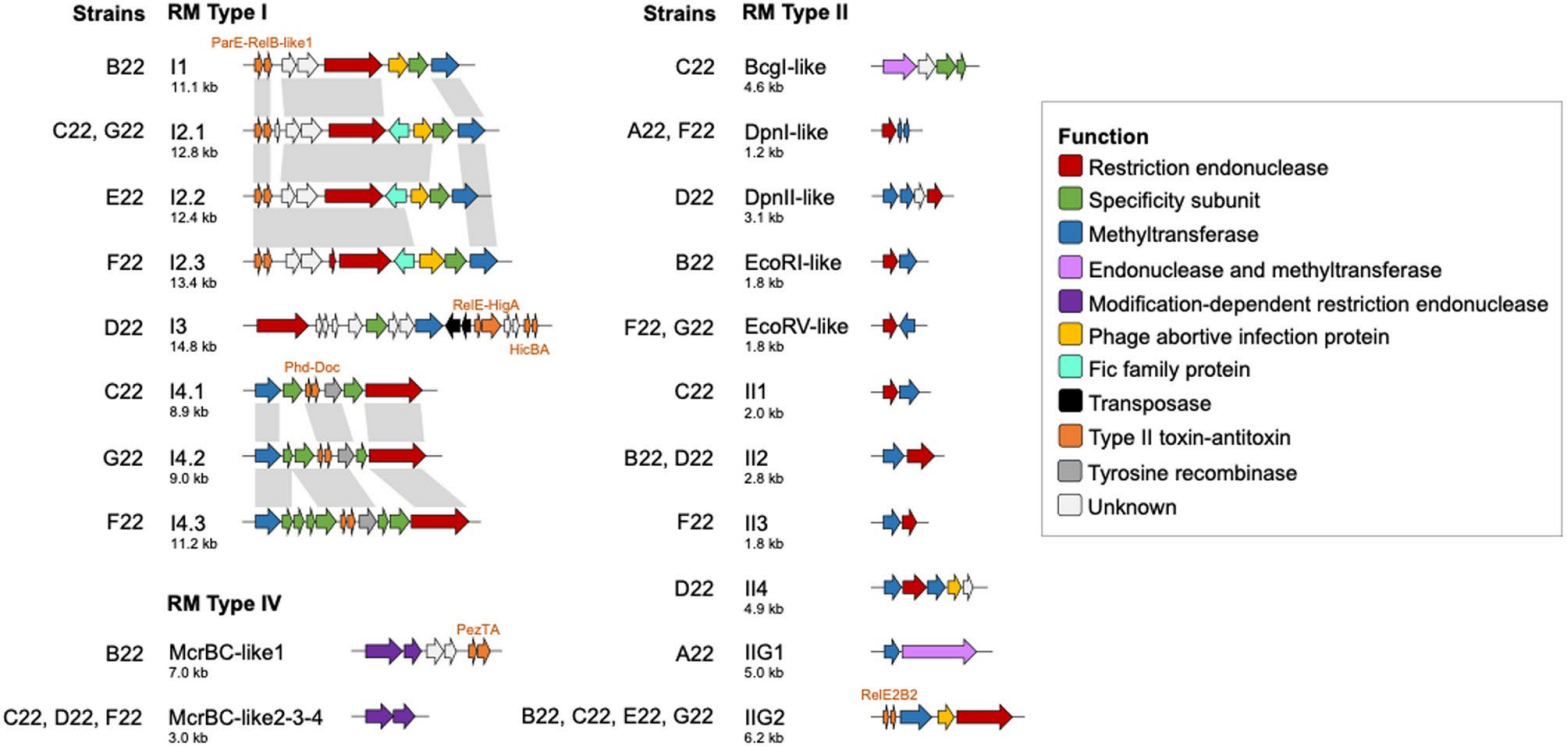
Restriction-modification (RM) systems found among the commensal streptococcal study strains. Type II toxin-antitoxin clusters encoded within or in the vicinities of the RM systems are also represented. The locus tags correspondent to the represented RM systems are indicated in Table S5A

Restriction-modification (RM) systems are bacterial defense mechanisms against foreign DNA, also playing a role in stabilization of genomic islands and recombination regions (reviewed in [51]). They comprise restriction endonuclease activity, that recognizes and cleaves foreign DNA at specific sites, and methyltransferase activity, that methylates the same sites (cytosine or adenine) in the host genome to distinguish foreign from self-DNA. Rather than acting as strict barriers, RM systems can influence how bacteria acquire new traits by selecting compatible foreign DNA and facilitating recombination events. We identified 23 putative RM systems (eight type I, 12 type II, and four type IV, classified based on genetic organization and sequence similarity of specificity subunits or MTase enzymes) in the genomes of the commensal study strains (Fig. 1C). *S. oralis* A22 encoded one RM system, while *S. mitis* strains encoded three to six (Fig. 1C and Table S5A). Five type II and the four type IV systems resembled known systems: BcgI (*Bacillus coagulans*), DnpI and DpnII (*S. pneumoniae*), EcoRI, EcoRV, and McrBC (*Escherichia coli*). Of note, the *dpnD* methyltransferase gene in system DpnI-like had a frameshift mutation resulting in two coding sequences that, nonetheless, covered the total length predicted for *dpnD*. The remaining 14 systems are novel. BLASTp analyses revealed that the RM systems identified, in addition to being widespread in *S. mitis* and *S. oralis*, were also commonly found in *S. pseudopneumoniae* and *S. suis*, among other streptococcal species (Table S7A). Some of them were also found in non-streptococci. Interestingly, I4 systems had multiple specificity subunits, likely allowing for functional substitution to target different recognition sequences (Fig. 6). Moreover, six RM systems encoded phage abortive infection proteins, and ten systems (including all of type I) encoded also for toxin-antitoxin pairs within or near the loci (Fig. 6).

Fifteen putative type II toxin-antitoxin (TA) systems were identified (Fig. 1D). Type II TA systems are associated with plasmid post-segregational killing, persistence, antibiotic tolerance, programmed cell death, and stabilization of mobile elements. These systems are widespread due to their frequent association with mobile elements (reviewed in [52]). They comprise a stable toxin protein that, under favorable conditions, is neutralized in a harmless complex by a labile antitoxin protein. Under triggering conditions, the antitoxin is degraded, allowing the toxin to act on its cellular target. The *S. oralis* strain A22 encoded one TA system, whereas *S. mitis* strains encoded four to nine (Fig. 1D and S5B). BLASTp searches showed that seven of the 15 systems were common in *S. pneumoniae*, while the remaining eight were rare (Table S7B), in alignment with the existing literature [53]. Overall, most systems were mainly found in *S. mitis*, *S. oralis*, *S. pseudopneumoniae*, and *S. suis*, and less frequently in other non-pneumococcal streptococci. Interestingly, MazFE-like (strain E22) and RnlAB (strain G22) were nearly absent from streptococci (Table S7B). Six systems (HicBA, ParE-RelB-like1, PezTA in strain B22, Phd-Doc, RelE2B2, and RelE-HigA) were within or near RM systems (Fig. 6); one (PezTA in strain C22) was in a transposon, and one (MazFE in strains B22, C22, F22, and G22, and RnlAB) was in a prophage region. To the best of our knowledge, our study is the first to report such a diversity of type II toxin-antitoxin systems in *S. mitis*, of which only half are typically found among pneumococci.

Reinforcing this notion of genomic plasticity, two conjugative transposons were found among the study commensals: Tn*916* in strains B22, D22, and F22, and Tn*5253* in strain C22. Both types of transposons have been previously described among *Streptococcus* species, including *S. pneumoniae* [54, 55].

Taken together, our results demonstrate the diverse nature of restriction-modification and type II toxin-antitoxin systems found in commensal streptococci, which highlights their proneness to horizontal gene transfer. Such systems can impact the relative fitness, evolution, and survival of the species.

### Commensal streptococci exhibit a diverse set of prophages

**Fig. 7:**
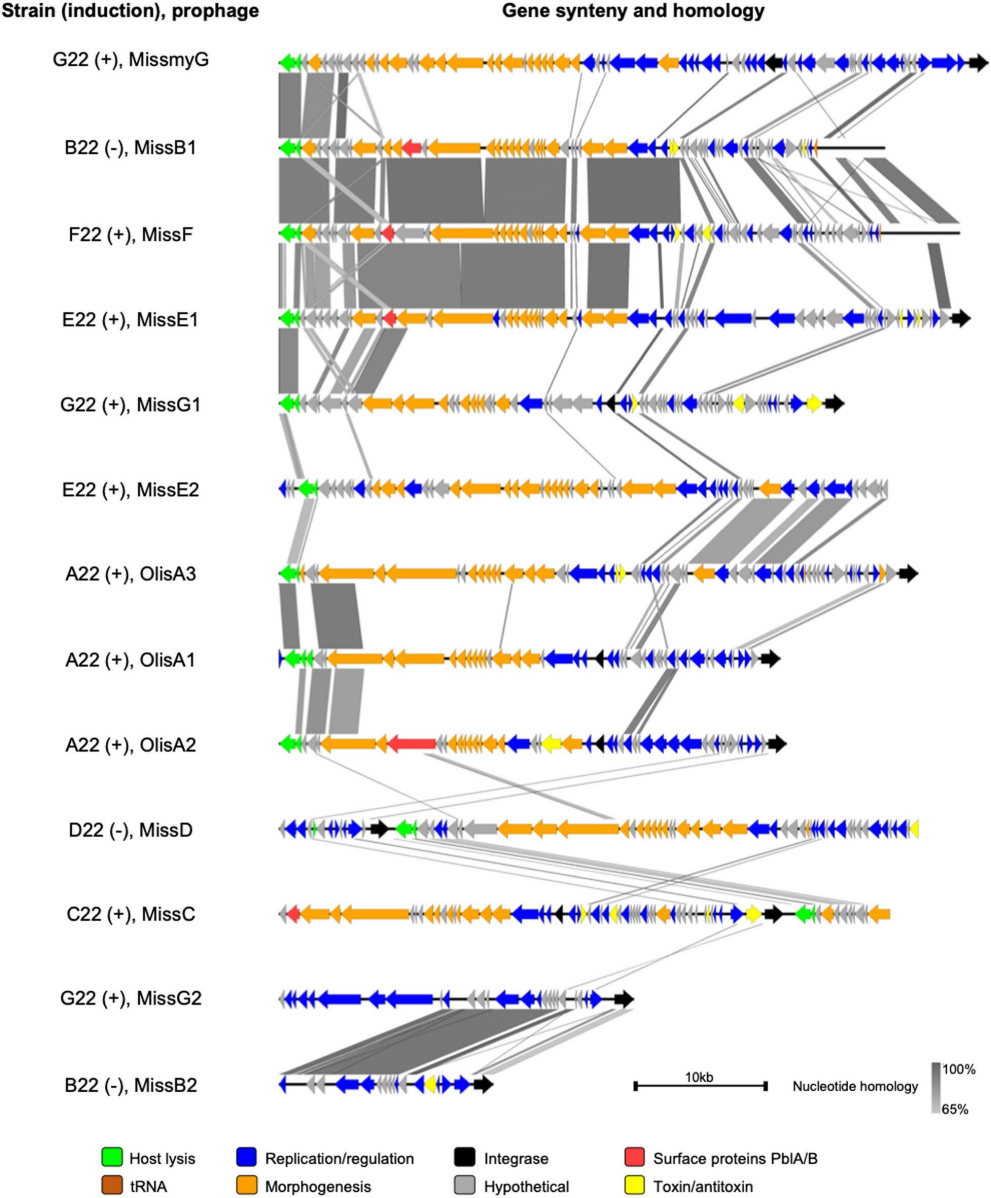
Genetic organization and synteny of prophages from the commensal streptococcal study strains. The 13 prophages are overall distinct between themselves, except for MissB1, MissF, and MissE1 that shared significant nucleotide homology in the region encoding morphogenic proteins. When treated with mitomycin C (a DNA cross-linking agent that triggers the SOS response and activates the lytic cycle of phages), culture lysis was observed for strains A22, C22, E22, F22, and G22 (indicated by (+)), whereas the growth of strains B22 and D22 were not significantly affected (indicated by (-)). The mitomycin C assay growth curves are depicted in Fig S4

We identified 13 distinct prophage regions in the genomes of the commensal study strains. Prophages are lysogenic phages that integrate in the bacterial genome and remain dormant until later activation (reviewed in [56]). Satellite prophages lack genes for their own replication, thus relying on “helper” prophages to replicate (reviewed in [57]). Eleven of the 13 prophages had genes for lysogeny, replication, packaging/morphology, and host lysis, while two were satellite prophages lacking self-replication genes (Fig. 7). Their genomes ranged from ca. 13 to 43 kb with 17 to 75 coding sequences and GC content ranging from 38.5 to 41.7% (Table 2). All prophages but one belonged to the Siphoviridae family; MismyG belonged to the Myoviridae. Phylogenetic analysis of the large terminase subunit showed six prophages using a headful packaging system and five using a cos mode (Table 2) [58, 59]. Mitomycin C induction activated all prophages except three (MissB1, MissB2, and MissD) (Fig. S4). Notably, eight prophages encoded a *lytA* allele (Fig. 7): *S. mitis* prophages shared up to 89% sequence identity with pneumococcal LytA, whereas *S. oralis* variants were up to 63% identical. Comparative genomics by BLASTn of full-length phage regions revealed low homology between most prophages and publicly available sequences, with closest homologues found in human oral metagenomes (Table 2) [60].

**Table 2:** Genomic features of the 13 prophages found amongst the study strains

Prophage	Strain	Size (bp)	CDS	Taxonomy	% GC	Packaging system	Closest homologue^b^ (% cover, % nt identity)
OlisA1	A22	30,210	49	Siphoviridae	39.8	Cos	BK039029 (82, 93)
OlisA2	A22	30,592	48	Siphoviridae	39.6	Cos	BK040408 (75, 94)
OlisA3	A22	38,516	65	Siphoviridae	38.5	Cos	BK057235 (46, 88)
MissB1	B22	36,522	68	Siphoviridae	41.7	Headful	BK040351 (25, 93)
MissB2^a^	B22	12,916	17	Siphoviridae	39.3	-	MK448645 (57,93)
MissC	C22	36,804	64	Siphoviridae	41.1	Cos	BK015441 (57, 94)
MissD	D22	38,511	62	Siphoviridae	38.6	Headful	BK059072 (63, 98)
MissE1	E22	41,671	66	Siphoviridae	41.4	Headful	BK039550 (74, 92)
MissE2	E22	36,665	62	Siphoviridae	39.9	Headful	BK045469 (53, 93)
MissF	F22	41,035	75	Siphoviridae	41.0	Headful	MK448908 (31, 94)
MismyG	G22	42,739	72	Myoviridae	39.6	Headful	BK037275 (45, 95)
MissG1	G22	34,071	63	Siphoviridae	40.8	Cos	BK057072 (41, 93)
MissG2^a^	G22	21,387	26	Siphoviridae	40.6	-	AP023349 (47, 96)

^a^ Satellite prophages ^b^ GenBank accession number

These findings highlight the novelty of these phages and their contribution to bacteriophage diversity in commensal streptococci.

### Commensal streptococci display distinct capsule-like morphologies

**Fig. 8:**
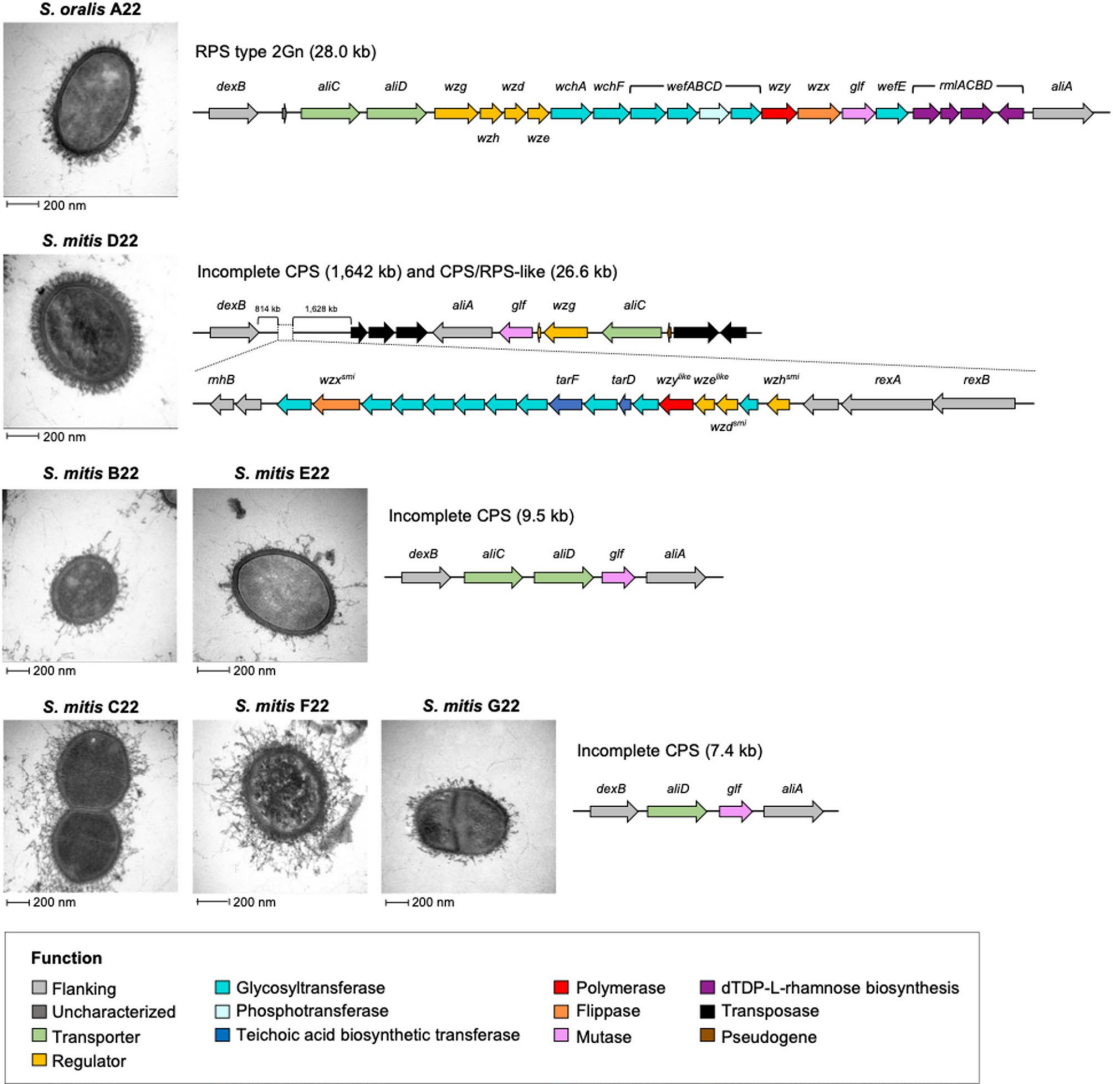
Transmission electron microscopy (TEM) images of the commensal streptococcal study strains and their genetic organization in the region homologous to the pneumococcal polysaccharide capsule. TEM images of strains A22, D22, E22, and F22 were taken at 87,000x magnification; whereas those of strains B22, C22, and G22 were taken at 60,000x magnification. The represented gene clusters correspond to the following locus tags: SORA22_16960 to SORA22_16740 (*S. oralis* A22); SMID22_19450 to SMID22_03100; zoom in SMID_11360 to SMID_11150 (*S. mitis* D22); SMIB22_17000 to SMIB22_16970 (*S. mitis* B22); SMIE22_16620 to SMIE22_16580 (*S. mitis* E22); SMIC22_17180 to SMIC22_17150 (*S. mitis* C22); SMIF22_17270 to SMIF22_17240 (*S. mitis* F22); and SMIG22_17870 to SMIG22_17840 (*S. mitis* G22)

The genomes of the seven commensal study strains were inspected between *dexB* and *aliA* genes for capsule loci. The capsular polysaccharide (CPS) is the main virulence factor of *S. pneumoniae*, with over 100 serotypes identified [61, 62]. Several Mitis group streptococci produce extracellular polysaccharides, from simple glucans to more complex structures [63–67]. The latter include the coaggregation receptor polysaccharide (RPS), which mediates physical interbacterial interactions in oral biofilms [64, 68]. Both CPS and RPS are synthesized via a Wzx/Wzy-dependent pathway [65, 69, 70]. We identified a complete RPS locus in *S. oralis* A22 (Fig. 8), encoding glycosyltransferases characteristic of type 2Gn RPS, previously found in *S. gordonii* [71]. Additionally, four genes encoding thymidine diphosphate-L-rhamnose (dTDP-L-Rha, an activated nucleotide sugar form of rhamnose that is incorporated as branches in the RPS) biosynthesis were present immediately upstream of *aliA*, a feature distinct from *S. gordonii* where these genes are typically located elsewhere in the chromosome due to a transposase in the end of the RPS locus [64]. 

In contrast, all *S. mitis* strains had incomplete CPS loci (Fig. 8). These loci encoded oligopeptide ABC transporters AliC and/or AliD and UDP-galactopyranose mutase Glf. Additionally, strain D22 encoded the transcriptional activator Wzg and a fragment of the regulatory protein Wzh (Fig. 8). Notably, the incomplete CPS locus in strain D22 was flanked by transposases, and *aliA* was positioned far away (approximately 1,630 kb downstream of *dexB*) from its usual location, representing a clear event of chromosomal rearrangement. Due to this atypical genomic disposition, we searched for capsule genes elsewhere in strain D22 genome and found a hybrid and nearly complete CPS/RPS-like locus approximately 814 kb downstream of *dexB* (Fig. 8). This locus differed in gene organization, lacked the regulatory *wzg* gene, and encoded putative cell wall-associated teichoic acid biosynthesis transferases. BLASTp searches revealed homologues of nearly all genes restricted to *S. mitis* strains. Notably, the *wzg* gene in the incomplete CPS locus in strain D22 could, in principle, functionally complement the missing one in its CPS/RPS-like locus.

Transmission electron microscopy (TEM) revealed distinct capsule-like morphologies in the study strains (Fig. 8). *S. oralis* strain A22 and *S. mitis* strain D22 showed a denser and structured capsule layer, in agreement with the genomic findings of complete capsule-like operons in these strains. Strain D22 had a uniform, tight layer, with capsule strands perpendicular to the cell wall, distinct from known streptococcal capsules. The other five study strains displayed sparce, “hairy” projections, possibly due to other polysaccharides and/or proteins [72, 73].

Taken together, our results highlight both the genetic and morphological diversity of commensal streptococci polysaccharide structures, suggesting distinct immunogenic properties.

### Commensal streptococci are structurally diverse in their cell wall teichoic acid composition

**Fig. 9:**
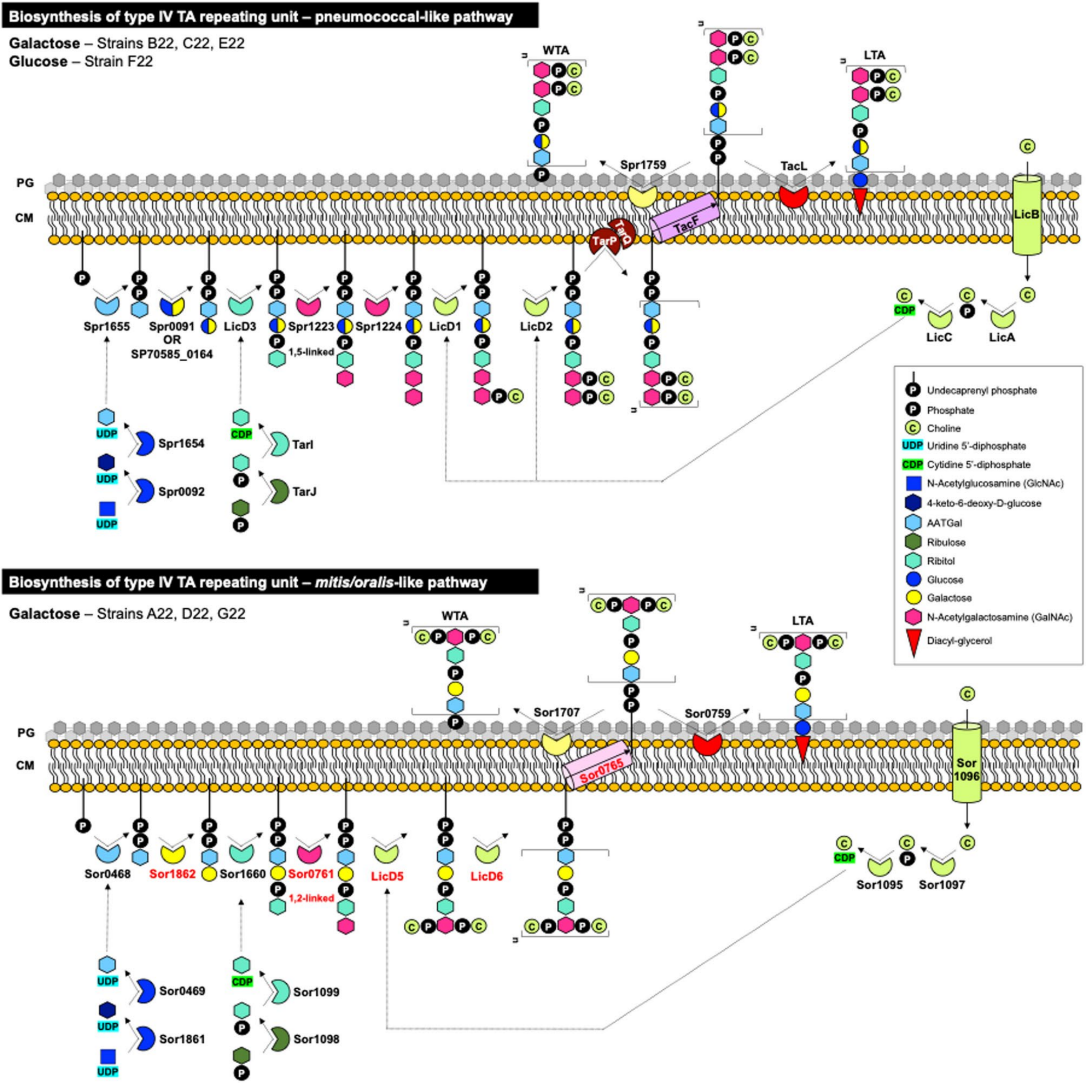
Biosynthetic pathways for teichoic acid repeating units in the commensal streptococcal study strains. The study strains B22, C22, E22, and F22 had genes encoding proteins similar to those of the pneumococcal teichoic acid pathway. Strain F22 was predicted to incorporate glucose; strains B22, C22 and E22 were predicted to incorporate galactose. Strains A22, D22, and G22 had genes encoding proteins similar to those of the *S. oralis* pathway. PG represents peptidoglycan; CM represents cytoplasmic membrane. Adapted from [78]. The locus tags correspondent to the represented pathways are indicated in Table S5C

The genomes of the seven commensal study strains were examined for genes involved in teichoic acid biosynthetic pathways. Teichoic acids are key cell wall components of Gram-positive bacteria, with pneumococcal lipoteichoic acids (type IV LTA, choline-containing) being the most complex described to date [74, 75]. We identified different biosynthetic pathways among the study strains. *S. oralis* strain A22 and *S. mitis* strains D22 and G22 likely produce type IV teichoic acids, previously described for *S. oralis* strains [76], which differ from pneumococcal versions in the number and type of carbohydrate moieties, linkage between residues, and phosphorylcholine substitution pattern (Fig. 9 and Table S5C). Notably, these strains incorporate galactose instead of glucose in their repeating units. In contrast, *S. mitis* strains B22, C22, E22, and F22 are predicted to produce pneumococcal-like type IV teichoic acids, with the first three strains incorporating galactose and F22 incorporating glucose (Fig. 9 and Table S5C). All *S. mitis* strains encoded the LTA synthase LtaS (97% identity to *S. mitis* B6 LtaS), essential for type I LTA synthesis [77]. Examination of additional genomes from the NCBI database (Table S1C) confirmed these patterns: four *S. mitis* and all *S. oralis* strains had *S. oralis*-like biosynthesis pathways, while five *S. mitis* exhibited the galactose-pneumococcal-like pathway. These results support divergent teichoic acid compositions and structures between commensal streptococci, with *S. mitis* displaying intraspecies heterogeneity.

### Commensal streptococci have diverse two-component systems

Two-component systems (TCS) are widespread bacterial regulatory mechanisms that allow bacteria to sense and respond to environmental signals, aiding in nutrient acquisition, adaptation, defense, and DNA uptake [79, 80]. The pneumococcal genome encodes 13 conserved histidine kinase-response regulator pairs (TCS01-13) and an orphan regulator (TCS14) [81].

We found that the study strains harbor a diverse set of TCSs crucial for adaptation. Eleven pneumococcal TCSs (TCS01-03, 05, 08–14) were highly conserved across all study strains, while TCS04 present in pneumococci was absent, consistent with previous findings in non-pneumococcal streptococci (Figure S5, Table S5D). TCS06 was missing in *S. oralis* strain A and *S. mitis* strain D, while TCS07 was absent only in strain D.

Additionally, 11 novel non-pneumococcal TCSs (TCS15-25) were differentially distributed among strains. Several were linked to bacteriocin loci (TCS15, 17, 20–23), while others were associated with sugar (TCS18, 25), iron (TCS19), thiamine (TCS25), and polyamine (TCS16) metabolism. TCS16 was the only non-pneumococcal system universally present. Homology searches identified these TCSs in other commensal streptococci (Table S5D), suggesting evolutionary adaptation.

Overall, *S. mitis* and *S. oralis* share key regulatory mechanisms with *S. pneumoniae* but also possess unique TCSs that may enhance survival and emphasize their genomic plasticity.

## Discussion

**Fig. 10:**
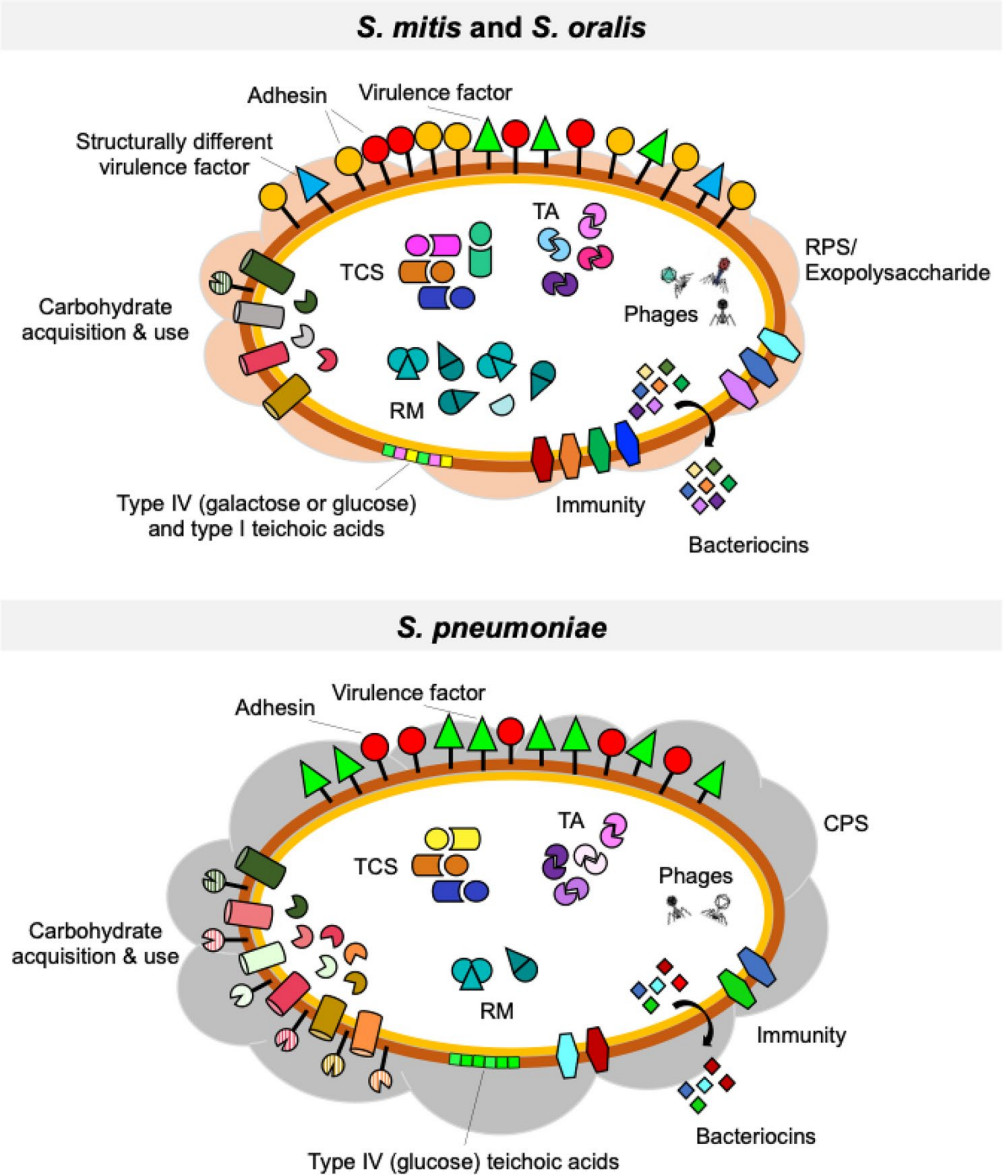
Schematic representation highlighting the key genomic differences between the commensal study strains *S. mitis* and *S. oralis* and pneumococci, as inferred from genomic analyses. The main genomic differences between the seven commensal streptococcal strains– chosen due to their anti-pneumococcal activity and thoroughly characterized in this study– and *S. pneumoniae* (extensively characterized over the years) are summarized. Major differences on surface proteins, bacteriocins [25], carbohydrate (degradation, uptake and use) machinery, antimicrobial resistance, restriction-modification systems, type II toxin-antitoxin systems, mobile elements (prophages), lipoteichoic acid composition, and the capsular polysaccharide are graphically summarized

In this study, we expanded our genomic analysis of seven commensal streptococcal strains belonging to *S. mitis* and *S. oralis*, previously identified as potential candidates for preventing pneumococcal colonization [25]. Despite the close phylogenetic relationship among *S. mitis*, *S. oralis*, and *S. pneumoniae*, key genomic differences likely underpin their distinct colonization and pathogenic potentials (Fig. 10).

Our analysis revealed that the study strains encode a higher number of putative surface proteins, particularly LPXTG-containing and choline-binding proteins, compared to pneumococci (Fig. 10). These proteins are frequently associated with bacterial adhesion and colonization, suggesting that commensal streptococci may rely on enhanced surface interactions to persist within the upper respiratory tract. In contrast, major pneumococcal virulence factors, including pilus islets, PcpA, PspA, PspC, and HysA, were absent from all commensal study strains. Even when homologues of pneumococcal virulence factors were present, they exhibited significant structural differences, which may alter their function and contribute to the commensal lifestyle.

Metabolic adaptations also distinguish these commensal strains from pneumococci (Fig. 10). We found that the commensals possessed a reduced repertoire of enzymes for acquiring carbohydrates from complex sources, suggesting a more limited ability to break down polysaccharides. However, the presence of transporters and intracellular enzymes targeting intermediate metabolites of these pathways - despite lacking the initial depolymerization enzymes - suggests a “cheater” strategy, whereby commensal streptococci exploit extracellular metabolites released by other microorganisms. Such cross-feeding relationship has been previously observed between several bacterial species, particularly in the human gut [82]. This opportunistic metabolic adaptation may be particularly relevant in the nutrient-scarce nasopharynx, where efficient nutrient acquisition is essential for bacterial survival. The ability of commensal streptococci to coexist with pneumococci could be facilitated by the latter’s extensive carbohydrate-active enzyme repertoire, allowing commensals to benefit from pneumococcal metabolic activity. A similar opportunistic relationship may also exist with other upper respiratory tract microorganisms equipped with diverse carbohydrate-degrading enzymes. Additionally, horizontal gene transfer appears to play a role in enhancing metabolic adaptability, as evidenced by the acquisition of additional transporter genes.

Another striking feature distinguishing the commensal strains from pneumococci is their extensive diversity of restriction-modification (RM) and type II toxin-antitoxin (TA) systems (Fig. 10). RM systems, which protect bacteria from foreign DNA, were highly variable among the commensal strains, with close homologues found not only in *S. mitis* and *S. oralis* but also in more distantly related species such as *S. pseudopneumoniae* and *S. suis*. This aligns with previous findings showing that RM systems in *S. mitis* and *S. oralis* vary significantly at the strain level [10]. The presence of diverse RM systems suggests that horizontal gene transfer has played a significant role in shaping the genetic repertoire of commensal streptococci, potentially influencing their adaptability and competitiveness within the upper respiratory tract. Likewise, the identified TA systems, which often function in stress response and genetic stability, may contribute to bacterial persistence.

We also identified 13 novel prophages within the commensal strains, expanding the known catalogue of prophages in these species. Across the broader Streptococcus genus, over 750 distinct prophages and satellite prophages have been identified, underscoring the extensive diversity of these mobile genetic elements [83]. Several TA systems in the study strains were located within or near RM gene clusters or prophage regions. Some RM systems encoded phage abortive infection (Abi) proteins, which induce premature cell death upon phage infection, limiting viral replication and spread [84]. Conversely, prophages impose selective pressures on their bacterial hosts, as phage survival depends on host fitness [85]. Notably, a recent study described phage small proteins that can modulate bacterial host gene expression [86]. To enhance their persistence, prophages often carry accessory genes that provide beneficial traits, such as RM and TA systems that confer resistance against subsequent phage infection [87, 88]. Notably, MazFE, found in five study strains and prophage-encoded in four, and RnlAB, identified in one strain, have both been described as phage inhibitors in *E. coli* [89, 90]. Together, RM systems, TA systems, and prophages may act synergistically to regulate phage susceptibility and enhance bacterial survival.

Capsular polysaccharide diversity further differentiates the commensal strains from pneumococci (Fig. 10). Two strains encoded complete capsule operons following the Wzx/Wzy-dependent biosynthetic pathway, with capsule production confirmed by transmission electron microscopy. This aligns with prior studies demonstrating that *S. mitis* and *S. oralis* express polysaccharide structures with immunochemical properties distinct from pneumococcal serotypes despite similarities in biosynthetic pathways [65]. To our best knowledge, our study is the first to report an *S. oralis* strain encoding a type 2Gn coaggregation receptor polysaccharide, which may facilitate interspecies biofilm formation - a trait consistent with the frequent detection of *S. oralis* in oral polymicrobial biofilms [91–93]. Additionally, *S. mitis* strain D22 exhibited a dense and structured capsule layer that appears distinct from known streptococcal capsules. In the five strains lacking complete capsule loci, scattered polymer-like structures were observed in transmission electron micrographs, resembling the “hairy” exopolysaccharides previously described in non-encapsulated *S. mitis* strains.

Beyond capsular variation, we observed significant differences in teichoic acid composition between commensal streptococci and pneumococci (Fig. 10). Biochemical analyses suggest that *S. oralis* strains produce type IV lipoteichoic acid (LTA) incorporating galactose instead of glucose in the repeating units, aligning with prior studies on LTA composition [76]. In contrast, *S. mitis* strains appeared to produce both type I and type IV LTA, similar to *S. suis* [94]. The predominance of galactose in LTA from *S. mitis* and *S. oralis*, corroborated by other studies [10, 76–78], contrasts with pneumococcal LTA, which primarily incorporates glucose. Given that galactose is the dominant carbohydrate in the nasopharynx while glucose prevails in the bloodstream, these structural differences may influence host interactions and bacterial adaptation to distinct niches. The greater structural diversity of teichoic acids in commensal streptococci compared to pneumococci may also have implications for immune recognition and could represent a potential anti-pneumococcal target [95, 96].

## Conclusions

Our study provides novel genomic insights into seven commensal *Streptococcus* strains with broad anti-pneumococcal activity recently described [25]. By identifying key genetic determinants contributing to their colonization and survival, we highlight significant differences between these commensal strains and the closely related pathogen *S. pneumoniae*. Our findings reinforce the role of horizontal gene transfer in shaping commensal bacterial genomes and emphasize the importance of bacterial characterization in identifying species-specific traits that may inform novel pathogen control strategies. Future experimental studies will be crucial in validating these genomic observations and elucidating the functional implications of the identified genetic adaptations.

## Electronic supplementary material

Below is the link to the electronic supplementary material.

Supplementary Material 1

Supplementary Material 2

## Data Availability

Reads and assembled annotated complete genomes of strains A22-G22 were deposited in European Nucleotide Archive (ENA) under the BioProject accession number PRJEB75690 (Table S3 includes direct links). Reads and annotated genomes of 13 prophages were deposited in NCBI under BioProject accession number PRJNA784746 (Table S3 includes direct links).

## Abbreviations

ABC: ATP-binding cassette
Abi: Phage abortive infection proteins
Cbp: Choline-binding proteins
CPS: Capsular polysaccharide
ENA: European Nucleotide Archive
LTA: Lipoteichoic acid
PTS: Phosphoenolpyruvate: sugar phosphotransferase systems
RM: Restriction-modification
RPS: Receptor polysaccharide
SMG: Streptococci of the Mitis group
TA: Toxin-antitoxin
TCS: Two-component systems
TEM: Transmission electron microscopy
URT: Upper respiratory tract
