# Supplementary material for: Genomic and functional insights into commensal streptococci with anti-pneumococcal activity
Source: BMC Genomics. 2025 Jul 1;26:577. doi: 10.1186/s12864-025-11756-x (PMC12211801; doi:10.1186/s12864-025-11756-x)
Supplement: Supplementary file 1 — Supplementary Material 1 [file 12864_2025_11756_MOESM1_ESM.pdf]

# SUPPLEMENTARY METHODS

## Method S1. BLAST analysis

All negative BLASTp matches (not a single hit in the BLASTp search) were manually confirmed by tBLASTn of the reference query protein sequences against the genome of the commensal streptococcal strains. All positive matches were manually inspected (sequence length and alignment with query) and confirmed by BLASTp against the NCBI non-redundant database. Matches covering ≥90% query length and sharing ≥80% amino acid identity with the reference were considered true positives. Whenever necessary, and mandatory for matches covering <90% of query length and/or sharing <80% of amino acid identity with the reference, further confirmation was done by analyzing the protein family and conserved domains using several tools and databases (using default parameters): NCBI Conserved Domain Search tool (1), InterProScan tool (2), SMART tool (3), PFAM database 34.0 (4), and PROSITE database (5). When appropriate, protein signal peptides and their respective cleavage sites were predicted using the tool SignalP-5.0 (6). Whenever necessary, a general search for similar hits in other bacterial species was done using UniProt (7). Overall, matches were considered true positives if the information gathered using the strategies above was concordant and trustful.

## Method S2. Phylogenetic analysis of choline-binding proteins

An alignment of 178 choline-binding protein (Cbp) sequences was done using Qiagen CLC Genomics Workbench software v9.5.1 (Qiagen, Venlo, The Netherlands) with default parameters: gap open cost of 10.0; gap extension cost of 1.0; end gap cost as any other; very accurate alignment mode. A neighbor-joining phylogenetic tree was generated based on the Cbp alignment using the Jukes-Cantor model for protein distance measure and bootstrap analysis based on 500 replicates (8). The protein sequences were retrieved from the complete genomes of the seven study strains (131 Cbp sequences) and from complete genomes of 15 *S. pneumoniae*, 10 *S. mitis,* and 16 *S. oralis* strains deposited at NCBI database (47 Cbp sequences).

**References**


1. Lu S, Wang J, Chitsaz F, Derbyshire MK, Geer RC, Gonzales NR, Gwadz M, Hurwitz DI, Marchler GH, Song JS, Thanki N, Yamashita RA, Yang M, Zhang D, Zheng C, Lanczycki CJ, Marchler-Bauer A. 2020. CDD/SPARCLE: the conserved domain database in 2020. Nucleic Acids Res 48:D265-D268.

2. Paysan-Lafosse T, Blum M, Chuguransky S, Grego T, Pinto BL, Salazar GA, Bileschi ML, Bork P, Bridge A, Colwell L, Gough J, Haft DH, Letunić I, Marchler-Bauer A, Mi H, Natale DA, Orengo CA, Pandurangan AP, Rivoire C, Sigrist CJA, Sillitoe I, Thanki N, Thomas PD, Tosatto SCE, Wu CH, Bateman A. 2023. InterPro in 2022. Nucleic Acids Res 51:D418-D427.

3. Letunic I, Khedkar S, Bork P. 2021. SMART: recent updates, new developments and status in 2020. Nucleic Acids Res 49:D458-D460.

4. Mistry J, Chuguransky S, Williams L, Qureshi M, Salazar GA, Sonnhammer ELL, Tosatto SCE, Paladin L, Raj S, Richardson LJ, Finn RD, Bateman A. 2021. Pfam: The protein families database in 2021. Nucleic Acids Res 49:D412-D419.

5. Sigrist CJ, Cerutti L, de Castro E, Langendijk-Genevaux PS, Bulliard V, Bairoch A, Hulo N. 2010. PROSITE, a protein domain database for functional characterization and annotation. Nucleic Acids Res 38:D161-6.

6. Almagro Armenteros JJ, Tsirigos KD, Sønderby CK, Petersen TN, Winther O, Brunak S, von Heijne G, Nielsen H. 2019. SignalP 5.0 improves signal peptide predictions using deep neural networks. Nat Biotechnol 37:420-423.

7. Consortium U. 2021. UniProt: the universal protein knowledgebase in 2021. Nucleic Acids Res 49:D480-D489.

8. TH J, CR C. 1969. Evolution of protein molecules. In Mammalian Protein Metabolism. Academic Press, New York.

## SUPPLEMENTARY TABLES

**Table S1. Pneumococcal genes and proteins used as queries for BLAST search in the commensal study strains.** All queries belong to *S. pneumoniae* strains except for Gtf4, LicD4, Flp, Gtf5, and LtaS (teichoic acid biosynthesis) which are from *S. mitis* B6 and S. *oralis* Uo5 strains. CPS – Capsule polysaccharide; RM – Restriction-modification; TA – toxin-antitoxin.

| Class | Protein | Strain | Locus tag | Protein ID | Size (aa) |
|---|---|---|---|---|---|
| LPXTG proteins | BgaA | TIGR4 | SP_0648 | AAK74795.1 | 2233 |
| | DiiA | TIGR4 | SP_1992 | AAK76059.1 | 221 |
| | EndoD | TIGR4 | SP_0498 | AAK74656.1 | 1659 |
| | HysA | TIGR4 | SP_0314 | AAK74491.1 | 1066 |
| | MucB | TIGR4 | SP_1492 | AAK75584.1 | 202 |
| | PavB | TIGR4 | SP_0082 | AAK74270.1 | 857 |
| | PfbA | TIGR4 | SP_1833 | AAK75906.1 | 708 |
| | PrtA | TIGR4 | SP_0641 | AAK74791.1 | 2140 |
| | PsrP | TIGR4 | SP_1772 | AAK75846.1 | 4776 |
| | RrgB | TIGR4 | SP_0463 | AAK74623.1 | 665 |
| | RrgC | TIGR4 | SP_0464 | AAK74624.1 | 393 |
| | SpGH101 | TIGR4 | SP_0368 | ABC75807.1 | 1767 |
| | SpuA | TIGR4 | SP_0268 | AAK74446.1 | 1280 |
| | ZmpA | TIGR4 | SP_1154 | AAK75263.1 | 2004 |
| | ZmpB | TIGR4 | SP_0664 | AAK74809.1 | 1881 |
| | ZmpC | TIGR4 | SP_0071 | AAK74260.1 | 1856 |
| | ZmpD | JJA | SPJ1074 | ACO20032.1 | 1737 |
| | PitA | INV104 | INV104_08690 | CBW36552.1 | 589 |
| | PitB | INV104 | INV104_08710 | CBW36554.1 | 410 |
| | NanA | R6 | spr1536 | AAL00340.1 | 1035 |
| | PclA | R6 | spr1403 | AAL00207.1 | 2551 |
| | StrH | R6 | spr0057 | AAK98861.1 | 1312 |
| Choline-binding proteins | CbpC | TIGR4 | SP_0377 | AAK74544.1 | 340 |
| | CbpD | TIGR4 | SP_2201 | AAK76252.1 | 448 |
| | CbpE | TIGR4 | SP_0930 | AAK75054.1 | 627 |
| | CbpG | TIGR4 | SP_0390 | AAK74556.1 | 285 |
| | CbpI | TIGR4 | SP_0069 | AAK74258.1 | 211 |
| | CbpJ | TIGR4 | SP_0378 | AAK74545.1 | 332 |
| | CbpK | TIGR4 | SP_0391 | AAK74557.1 | 340 |
| | CbpL | TIGR4 | SP_0667 | AAK74812.1 | 332 |
| | LytA | TIGR4 | SP_1937 | AAK76005.1 | 318 |
| | LytB | TIGR4 | SP_0965 | AAK75086.1 | 658 |
| | LytC | TIGR4 | SP_1573 | ABC75800.1 | 490 |
| | PcpA | TIGR4 | SP_2136 | AAK76194.1 | 621 |
| | PspA | TIGR4 | SP_0117 | AAK74303.1 | 744 |
| | PspC | TIGR4 | SP_2190 | AAK76241.1 | 693 |
| | CbpM | R6 | spr1274 | AAL00078.1 | 129 |
| Lipoproteins | AatB | TIGR4 | SP_1500 | AAK75591.1 | 278 |
| | AdcA | TIGR4 | SP_2169 | AAK76223.1 | 501 |
| | AdcAII | TIGR4 | SP_1002 | AAK75119.1 | 305 |
| | AliA | TIGR4 | SP_0366 | AAK74534.1 | 661 |
| | AliB | TIGR4 | SP_1527 | AAK75616.1 | 652 |
| | AmiA | TIGR4 | SP_1891 | AAK75962.1 | 659 |
| | ComM | TIGR4 | SP_1945 | AAK76013.1 | 206 |
| | GlnH | TIGR4 | SP_1394 | AAK75492.1 | 271 |
| | GshT | TIGR4 | SP_0148 | AAK74330.1 | 276 |
| | LdcB | TIGR4 | SP_0629 | AAK74780.1 | 238 |
| | LivJ | TIGR4 | SP_0749 | AAK74888.1 | 386 |
| | MetQ | TIGR4 | SP_0149 | AAK74331.1 | 284 |
| | OxaA1 | TIGR4 | SP_2041 | AAK76106.1 | 274 |
| | OxaA2 | TIGR4 | SP_1975 | AAK76042.1 | 308 |
| | PiaA | TIGR4 | SP_1032 | AAK75147.1 | 341 |
| | PiuA | TIGR4 | SP_1872 | AAK75944.1 | 321 |
| | PiuC | TIGR4 | SP_1870 | AAK75942.1 | 318 |

**Table S1** (*cont.*)

| Class | Protein | Strain | Locus tag | Protein ID | Size (aa) |
|---|---|---|---|---|---|
| **Lipoproteins** (*cont.*) | PrsA (PpmA) | TIGR4 | SP_0981 | AAK75102.1 | 313 |
| | PsaA | TIGR4 | SP_1650 | AAK75729.1 | 309 |
| | PstS1 | TIGR4 | SP_1400 | AAK75498.1 | 292 |
| | PtsS2 | TIGR4 | SP_2084 | AAK76144.1 | 291 |
| | SlrA (PpiA) | TIGR4 | SP_0771 | AAK74909.1 | 267 |
| | SP_0112 | TIGR4 | SP_0112 | AAK74299.1 | 268 |
| | SP_0191 | TIGR4 | SP_0191 | AAK74372.1 | 189 |
| | SP_0198 | TIGR4 | SP_0198 | AAK74378.1 | 152 |
| | SP_0620 | TIGR4 | SP_0620 | AAK74772.1 | 266 |
| | SP_0659 | TIGR4 | SP_0659 | AAK74804.1 | 188 |
| | SP_0899 | TIGR4 | SP_0899 | AAK75025.1 | 290 |
| | SP_1000 | TIGR4 | SP_1000 | AAK75117.1 | 185 |
| | SP_1826 | TIGR4 | SP_1826 | AAK75899.1 | 355 |
| | SrtC-3 | TIGR4 | SP_0468 | AAK74628.1 | 283 |
| | ThiY | TIGR4 | SP_2197 | AAK76248.1 | 335 |
| **Non-classical surface exposed proteins** | 6PGD | TIGR4 | SP_0375 | AAK74542.1 | 474 |
| | CppA | TIGR4 | SP_1449 | AAK75543.1 | 241 |
| | Eno | TIGR4 | SP_1128 | AAK75238.1 | 434 |
| | Fic-like | TIGR4 | SP_0571 | AAK74726.1 | 267 |
| | GAPDH | TIGR4 | SP_2012 | AAK76079.1 | 335 |
| | HtrA | TIGR4 | SP_2239 | AAK76286.1 | 393 |
| | NanB | TIGR4 | SP_1687 | AAK75766.1 | 697 |
| | NanC | TIGR4 | SP_1326 | AAK75424.1 | 740 |
| | PavA | TIGR4 | SP_0966 | AAK75087.1 | 551 |
| | Pbp1B | TIGR4 | SP_2099 | AAK76158.1 | 821 |
| | PcsB | TIGR4 | SP_2216 | AAK76264.1 | 392 |
| | PhtA | TIGR4 | SP_1175 | AAK75284.1 | 802 |
| | PhtB | TIGR4 | SP_1174 | AAK75283.1 | 819 |
| | PhtD | TIGR4 | SP_1003 | AAK75120.1 | 839 |
| | PhtE | TIGR4 | SP_1004 | AAK75121.1 | 1039 |
| | Ply | TIGR4 | SP_1923 | AAK5991.1 | 471 |
| | PppA | TIGR4 | SP_1572 | AAK5659.1 | 178 |
| | SpGH92 | TIGR4 | SP_2145 | AAK76202.1 | 694 |
| | StkP | TIGR4 | SP_1732 | AAK75808.1 | 659 |
| | Tig | TIGR4 | SP_0400 | AAK4553.1 | 427 |
| | SrtA | R6 | spr1098 | AAK99901.1 | 247 |
| **Carbohydrate uptake** | PTS1 | TIGR4 | SP_0061 | AAK74250.1 | 158 |
| | | TIGR4 | SP_0062 | AAK74251.1 | 301 |
| | | TIGR4 | SP_0063 | AAK74252.1 | 271 |
| | | TIGR4 | SP_0064 | AAK74253.1 | 134 |
| | PTS2 | TIGR4 | SP_0248 | AAK74427.1 | 106 |
| | | TIGR4 | SP_0249 | AAK74428.1 | 102 |
| | | TIGR4 | SP_0250 | AAK74429.1 | 440 |
| | PTS3 | TIGR4 | SP_0282 | AAK74460.1 | 303 |
| | | TIGR4 | SP_0283 | AAK74461.1 | 267 |
| | | TIGR4 | SP_0284 | AAK74462.1 | 332 |
| | PTS4 | TIGR4 | SP_0305 | AAK74482.1 | 104 |
| | | TIGR4 | SP_0308 | AAK74485.1 | 104 |
| | | TIGR4 | SP_0310 | AAK74487.1 | 448 |
| | PTS5 | TIGR4 | SP_0321 | AAK74497.1 | 144 |
| | | TIGR4 | SP_0323 | AAK74499.1 | 163 |
| | | TIGR4 | SP_0324 | AAK74500.1 | 259 |
| | | TIGR4 | SP_0325 | AAK74501.1 | 272 |
| | PTS6 | TIGR4 | SP_0394 | AAK74558.1 | 589 |
| | | TIGR4 | SP_0396 | AAK74560.1 | 145 |
| | PTS7 | TIGR4 | SP_0474 | AAK74633.1 | 440 |
| | | TIGR4 | SP_0476 | AAK74635.1 | 114 |
| | | TIGR4 | SP_0478 | AAK74637.1 | 559 |

**Table S1** (*cont.*)

| Class | Protein | Strain | Locus tag | Protein ID | Size (aa) |
|---|---|---|---|---|---|
| **Carbohydrate uptake** (*cont.*) | PTS8 | TIGR4 | SP_0577 | AAK74731.1 | 612 |
| | PTS9 | TIGR4 | SP_0645 | AAK74792.1 | 165 |
| | | TIGR4 | SP_0646 | AAK74793.1 | 101 |
| | | TIGR4 | SP_0647 | AAK74794.1 | 491 |
| | PTS10 | TIGR4 | SP_0758 | AAK74896.1 | 726 |
| | PTS11 | TIGR4 | SP_0877 | AAK75004.1 | 650 |
| | PTS12 | TIGR4 | SP_1185 | AAK75294.1 | 563 |
| | | TIGR4 | SP_1186 | AAK75295.1 | 105 |
| | PTS13 | TIGR4 | SP_1617 | AAK75698.1 | 361 |
| | | TIGR4 | SP_1618 | AAK75699.1 | 108 |
| | | TIGR4 | SP_1619 | AAK75700.1 | 149 |
| | PTS14 | TIGR4 | SP_1684 | AAK75763.1 | 505 |
| | PTS15 | TIGR4 | SP_1722 | AAK75799.1 | 627 |
| | PTS16 | TIGR4 | SP_1884 | AAK75956.1 | 655 |
| | PTS17 | TIGR4 | SP_2022 | AAK76087.1 | 431 |
| | | TIGR4 | SP_2023 | AAK76088.1 | 102 |
| | | TIGR4 | SP_2024 | AAK76089.1 | 108 |
| | PTS18 | TIGR4 | SP_2036 | AAK76101.1 | 161 |
| | | TIGR4 | SP_2037 | AAK76102.1 | 93 |
| | | TIGR4 | SP_2038 | AAK76103.1 | 485 |
| | PTS19 | TIGR4 | SP_2129 | AAK76187.1 | 448 |
| | | TIGR4 | SP_2130 | AAK76188.1 | 94 |
| | PTS20 | TIGR4 | SP_2161 | AAK76215.1 | 266 |
| | | TIGR4 | SP_2162 | AAK76216.1 | 257 |
| | | TIGR4 | SP_2163 | AAK76217.1 | 156 |
| | | TIGR4 | SP_2164 | AAK76218.1 | 143 |
| | PTS21 | Hungary 19A-6 | SPH_1925 | ACA37243.1 | 451 |
| | | Hungary 19A-6 | SPH_1926 | ACA36919.1 | 105 |
| | | Hungary 19A-6 | SPH_1927 | ACA36309.1 | 105 |
| | | Hungary 19A-6 | SPH_1929 | ACA37567.1 | 420 |
| | PtsI | TIGR4 | SP_1176 | AAK75285.1 | 577 |
| | PtsH | TIGR4 | SP_1177 | AAK75286.1 | 87 |
| | ABC1 | TIGR4 | SP_0090 | AAK74277.1 | 319 |
| | | TIGR4 | SP_0091 | AAK74278.1 | 307 |
| | | TIGR4 | SP_0092 | AAK74279.1 | 491 |
| | ABC2 | TIGR4 | SP_0845 | AAK74976.1 | 350 |
| | | TIGR4 | SP_0846 | AAK74977.1 | 511 |
| | | TIGR4 | SP_0847 | AAK74978.1 | 352 |
| | | TIGR4 | SP_0848 | AAK74979.1 | 318 |
| | ABC3 | TIGR4 | SP_1681 | AAK75760.1 | 279 |
| | | TIGR4 | SP_1682 | AAK75761.1 | 298 |
| | | TIGR4 | SP_1683 | AAK75762.1 | 442 |
| | ABC4 | TIGR4 | SP_1688 | AAK75767.1 | 277 |
| | | TIGR4 | SP_1689 | AAK75768.1 | 294 |
| | | TIGR4 | SP_1690 | AAK75769.1 | 445 |
| | ABC5 | TIGR4 | SP_1796 | AAK75869.1 | 538 |
| | | TIGR4 | SP_1797 | AAK75870.1 | 305 |
| | | TIGR4 | SP_1798 | AAK75871.1 | 305 |
| | ABC6 | TIGR4 | SP_1895 | AAK75966.1 | 278 |
| | | TIGR4 | SP_1896 | AAK75967.1 | 296 |
| | | TIGR4 | SP_1897 | AAK75968.1 | 419 |
| | ABC7 | TIGR4 | SP_2108 | AAK76167.1 | 423 |
| | | TIGR4 | SP_2109 | AAK76168.1 | 435 |
| | | TIGR4 | SP_2110 | AAK76169.1 | 280 |
| | ABC8 | G54 | SPG_2105 | ACF56527.1 | 279 |
| | | G54 | SPG_2106 | ACF56054.1 | 309 |
| | | G54 | SPG_2107 | ACF55321.1 | 430 |
| | MsmK | TIGR4 | SP_1580 | AAK75666.1 | 376 |
| | Symporter | TIGR4 | SP_1328 | AAK75426.1 | 513 |
| | Facilitator | TIGR4 | SP_1491 | AAK75583.1 | 289 |
| | Facilitator | TIGR4 | SP_2184 | AAK76235.1 | 234 |

**Table S1** (*cont.*)

| Class | Protein | Strain | Locus tag | Protein ID | Size (aa) |
|---|---|---|---|---|---|
| Carbohydrate use | ***Complex N-Glycans*** | | | | |
| | SpGH38 | TIGR4 | SP_2143 | AAK76200.1 | 886 |
| | SpGH125 | | SP_2144 | AAK76201.1 | 426 |
| | ***Fucose type 1 operon*** | | | | |
| | FcsR | TIGR4 | SP_2168 | AAK76222.1 | 257 |
| | FcsK | TIGR4 | SP_2167 | AAK76221.1 | 467 |
| | FcsA | TIGR4 | SP_2166 | AAK76220.1 | 212 |
| | FcsU | TIGR4 | SP_2165 | AAK76219.1 | 147 |
| | Sp4GH95A | TIGR4 | SP_2160 | AAK76214.1 | 764 |
| | Sp4GH98 | TIGR4 | SP_2159 | AAK76213.1 | 1038 |
| | FcsI | TIGR4 | SP_2158 | AAK76212.1 | 588 |
| | FcsO | TIGR4 | SP_2157 | AAK76211.1 | 383 |
| | ***Fucose type 2 operon*** | | | | |
| | FcsR | Sp3-BS71 | CGSSp3BS71_10443 | EDK74357.1 | 251 |
| | Fcsk | Sp3-BS71 | CGSSp3BS71_10438 | EDK74356.1 | 467 |
| | Sp3GH29 | Sp3-BS71 | CGSSp3BS71_10418 | EDK74352.1 | 435 |
| | Sp3GH36A | Sp3-BS71 | CGSSp3BS71_10413 | EDK74351.1 | 550 |
| | Sp3GH36B | Sp3-BS71 | CGSSp3BS71_10408 | EDK74350.1 | 738 |
| | Sp3GH98 | Sp3-BS71 | CGSSp3BS71_10403 | EDK74349.1 | 1005 |
| | FcsA | Sp3-BS71 | CGSSp3BS71_10398 | EDK74348.1 | 242 |
| | FcsI | Sp3-BS71 | CGSSp3BS71_10393 | EDK74347.1 | 588 |
| | FcsO | Sp3-BS71 | CGSSp3BS71_10388 | EDK74346.1 | 383 |
| | ***Glycosaminoglycans*** | | | | |
| | UGL | TIGR4 | SP_0322 | AAK74498.1 | 396 |
| | ***Dietary glycans*** | | | | |
| | ScrH | TIGR4 | SP_1724 | AAK75801.1 | 484 |
| | BglA-1 | TIGR4 | SP_0303 | AAK74480.1 | 478 |
| | BglA-2 | TIGR4 | SP_0578 | AAK74732.1 | 471 |
| | Aga | TIGR4 | SP_1898 | AAK75969.1 | 720 |
| | FusH | TIGR4 | SP_1795 | AAK75868.1 | 439 |
| | ***Glycogen*** | | | | |
| | MalM | TIGR4 | SP_2107 | AAK76166.1 | 505 |
| | MalP | TIGR4 | SP_2106 | AAK76165.1 | 752 |
| | AG13 | TIGR4 | SP_1883 | AAK75955.1 | 541 |
| | AMY | TIGR4 | SP_1382 | AAK75480.1 | 484 |
| CPS | DexB | TIGR4 | SP_0342 | AAK74516.1 | 535 |
| | AliA | TIGR4 | SP_0366 | AAK74534.1 | 661 |
| RM systems | DpnC | TIGR4 | SP_1850 | AAK75922.1 | 254 |
| | DnpD | TIGR4 | SP_1849 | AAK75921.1 | 153 |
| | DnpM | 70585 | SP70585_1905 | ACO16777.1 | 284 |
| | DnpA | 70585 | SP70585_1904 | ACO16124.1 | 268 |
| | DnpB | 70585 | SP70585_1903 | ACO18002.1 | 288 |
| | R.DpnIII | ATCC 700669 | SPN23F18640 | CAR69626.1 | 496 |
| | M.DpnIII | ATCC 700669 | SPN23F18650 | CAR69627.1 | 421 |
| Type II TA systems | COG2856C | TIGR4 | SP_1934 | AAK76002.1 | 141 |
| | COG2856A | TIGR4 | SP_1935 | AAK76003.1 | 119 |
| | Xre | TIGR4 | SP_1936 | AAK76004.1 | 53 |
| | HicA | TIGR4 | SP_1787 | AAK75860.1 | 68 |
| | HicB | TIGR4 | SP_1786 | AAK78859.1 | 150 |
| | HigB | TIGR4 | SP_1143 | AAK75253.1 | 121 |
| | HigA | TIGR4 | SP_1144 | AAK75254.1 | 97 |
| | PezT | TIGR4 | SP_1051 | AKK75165.1 | 253 |
| | PezA | TIGR4 | SP_1050 | AAK75164.1 | 158 |
| | Phd | TIGR4 | SP_0889 | AAK75016.1 | 137 |
| | Doc | TIGR4 | SP_0888 | AAK75015.1 | 78 |

**Table S1** (*cont.*)

| Class | Protein | Strain | Locus tag | Protein ID | Size (aa) |
|---|---|---|---|---|---|
| **TA** (*cont.*) | RelE1 | TIGR4 | SP_0276 | AAK74454.1 | 92 |
| | RelB1 | TIGR4 | SP_0275 | AAK74453.1 | 87 |
| | RelE2 | TIGR4 | SP_1223 | AAK75329.1 | 84 |
| | RelB2 | TIGR4 | SP_1224 | AAK75330.1 | 100 |
| | YoeB | TIGR4 | SP_1740 | AAK75816.1 | 84 |
| | YefM | TIGR4 | SP_1741 | AAK75817.1 | 84 |
| **Two-component systems** | HK01 (GraS) | TIGR4 | SP_1632 | AAK75712.1 | 324 |
| | RR01 (GraR) | TIGR4 | SP_1633 | AAK75713.1 | 225 |
| | HK02 (VicK) | TIGR4 | SP_1226 | AAK75332.1 | 449 |
| | RR02 (VicR) | TIGR4 | SP_1227 | AAK75333.1 | 234 |
| | HK03 (LiaS) | TIGR4 | SP_0386 | AAK74553.1 | 331 |
| | RR03 (LiaR) | TIGR4 | SP_0387 | AAK74554.1 | 210 |
| | HK04 (PnpS) | TIGR4 | SP_2083 | AAK76143.1 | 443 |
| | RR04 (PnpR) | TIGR4 | SP_2082 | AAK76142.1 | 235 |
| | HK05 (CiaH) | TIGR4 | SP_0799 | AAK74936.1 | 444 |
| | RR05 (CiaR) | TIGR4 | SP_0798 | AAK74935.1 | 224 |
| | HK06 | TIGR4 | SP_2192 | AAK76243.1 | 443 |
| | RR06 | TIGR4 | SP_2193 | AAK76244.1 | 217 |
| | HK07 (YesM) | TIGR4 | SP_0155 | AAK74337.1 | 548 |
| | RR07 (YesN) | TIGR4 | SP_0156 | AAK74338.1 | 428 |
| | HK08 (SaeS) | TIGR4 | SP_0084 | AAK74272.1 | 350 |
| | RR08 (SaeR) | TIGR4 | SP_0083 | AAK74271.1 | 232 |
| | HK09 (ZmpS) | TIGR4 | SP_0662 | AAK74807.1 | 563 |
| | RR09 (ZmpR) | TIGR4 | SP_0661 | AAK74806.1 | 245 |
| | HK10 (VncS) | TIGR4 | SP_0604 | AAK74756.1 | 442 |
| | RR10 (VncR) | TIGR4 | SP_0603 | AAK74755.1 | 218 |
| | HK11 (DesK) | TIGR4 | SP_2001 | AAK76068.1 | 365 |
| | RR11 (DesR) | TIGR4 | SP_2000 | AAK76067.1 | 199 |
| | HK12 (ComD) | TIGR4 | SP_2236 | AAK76283.1 | 441 |
| | RR12 (ComE) | TIGR4 | SP_2235 | AAK76282.1 | 250 |
| | HK13 (BlpH) | TIGR4 | SP_0527 | AAK74685.1 | 446 |
| | RR13 (BlpR) | TIGR4 | SP_0526 | AAK74684.1 | 245 |
| | RR14 (AlrR) | TIGR4 | SP_0376 | AAK74543.1 | 229 |
| **Teichoic acid biosynthesis** | LicC | R6 | spr1145 | AAK99948.1 | 229 |
| | LicB | R6 | spr1146 | AAK99949.1 | 292 |
| | LicA | R6 | spr1147 | AAK99950.1 | 262 |
| | TarJ | R6 | spr1148 | AAK99951.1 | 340 |
| | TarI | R6 | spr1149 | AAK99952.1 | 235 |
| | TacF | R6 | spr1150 | AAK99953.1 | 495 |
| | LicD1 | R6 | spr1151 | AAK99954.1 | 267 |
| | LicD2 | R6 | spr1152 | AAK99955.1 | 269 |
| | TarQ | R6 | spr1221 | AAL00025.1 | 344 |
| | TarP | R6 | spr1222 | AAL00026.1 | 477 |
| | Cps23FU | R6 | spr1223 | AAL00027.1 | 328 |
| | Gtf1 | R6 | spr1224 | AAL00028.1 | 367 |
| | LicD3 | R6 | spr1225 | AAL00029.1 | 281 |
| | TacL | R6 | spr1708 | AAL00511.1 | 397 |
| | Gtf2 | R6 | spr0091 | AKK98895.1 | 385 |
| | AatA | R6 | spr0092 | AKK98896.1 | 616 |
| | AatB | R6 | spr1654 | AAL00457.1 | 408 |
| | Trf | R6 | spr1655 | AAL00458.1 | 230 |
| | Trf | R6 | spr1759 | AAL00562.1 | 338 |
| | Gtf3 | 70585 | SP70585_0164 | ACO16938.1 | 276 |
| | Gtf4 | Uo5 (*S. oralis*) | SOR_0761 | WP_041170809.1 | 321 |
| | LicD4 | Uo5 (*S. oralis*) | SOR_0762 | WP_000622810.1 | 717 |
| | Flp | Uo5 (*S. oralis*) | SOR_0765 | WP_000789712.1 | 492 |
| | Gtf5 | Uo5 (*S. oralis*) | SOR_1862 | WP_001114305.1 | 276 |
| | LtaS | B6 (*S. mitis*) | smi_0753 | YP_003445868.1 | 716 |

**Table S2. Conserved domains used to identify additional choline-binding proteins, carbohydrate uptake transporters, type II toxin-antitoxin (TA) systems, and two-component systems (TCS) in the commensal study strains.**

| Class | | Domain | Domain name |
|---|---|---|---|
| **Choline-binding proteins** | | COG5263<br>PF01473<br>PF19127 | Glucan-binding<br>CW_binding_1<br>Choline_bind_3 |
| **Carbohydrate uptake transporters** | PTS Glucose Type | CD00210<br>CD00212<br>PF02378 | (PTS_IIA_Glc)<br>PTS_IIB_Glc<br>PTS_EIIC |
| | PTS Fructose Type | CD00133<br>CD00211<br>PF02378 | PTS_IIB<br>PTS_IIA_Fru<br>PTS_EIIC |
| | PTS Lactose Type | CD00215<br>CD05565<br>PF02378 | PTS_IIA_Lac<br>PTS_IIB_Lac<br>PTS_EIIC |
| | PTS Galactitol Type | CD00133<br>PF00359<br>PF03611 | PTS_IIB<br>PTS_EIIA_2<br>EIIC-GAT |
| | PTS Mannose Type | PF03609<br>PF03610<br>PF03613<br>PF03830 | EII-Sor<br>EIIA-man<br>EIID-AGA<br>PTSIIB_sorb |
| | PTS L-Ascorbate Type | CD05563<br>PF03611 | PTS_IIB_Asc<br>EIIC-GAT |
| | ABC CUT1 Permease | CD06261<br>PF00528 | TM_PBP2<br>BPD_Transp_1 |
| | ABC CUT1 Substrate-binding | CD14750<br>PF01547 | PBP2_TMBP<br>SBP_Bac_1 |
| | ABC CUT2 Permease | PF02653 | BPD_Transp_2 |
| | ABC CUT2 | CD00267<br>PF00005 | ABC_ATPase<br>ABC_Tran |
| **Type II TA systems** | BrnT/ BrnA | PF04365/ PF14384 | BrnT_toxin/ BrnA_antitoxin |
| | HicA/ HicB<br>HicB-like | PF07927/ PF05534<br>PF15919, PF15970 | HicA_toxin/ HicB<br>HicB_lk_antitox/ HicB-like_2 |
| | HigB/ HigA<br>HigB-like | PF09907/ COG5499<br>PF05015 | HigB_toxin/ HigA<br>HigB-like_toxin |
| | MazF/ MazE | PF02452/ PF04014 | PemK_toxin/ MazE_antitoxin |
| | MqsR/ MqsA | PF15723/ PF15731 | MqsR_toxin/ MqsA_antitoxin |
| | ParE/ ParD<br>ParD-like | PF05016/ PF03693<br>PF11903 | ParE_toxin/ ParD_antitoxin<br>ParD_like |
| | RelE/ RelB | PF06296/ PF04221 | RelE/ RelB |
| | RlnA/ RnlB | PF19034/ PF15933 | RnlA-toxin_DBD/ RnlB_antitoxin |
| | SpoIISA/ SpoIISB | PF14171/ PF14185 | SpoIISA_toxin/ SpoIISB_antitox |
| | YdaT/ YdaS | PF06254 / PF15943 | YdaT_toxin/ YdaS_antitoxin |
| | YoeB/ YefM | PF06769/ PF02604 | YoeB_toxin/ PhdYeFM_antitox |
| **TCS** | DNA-binding regulator | CD00156<br>CD17533 | REC<br>REC_LytTR_AgrA-like |
| | Histidine kinase | PF02518<br>PF06580<br>PF14501<br>CD16917 | HATPase_c<br>His_kinase<br>HATPase_c_5<br>HATPase_UhpB-NarQ-NarX-like |

**Table S3. Accession numbers of sequences obtained in this study and deposited in public databases.**

| Deposited in European Nucleotide Archive (ENA) database | | | |
|---|---|---|---|
| **Description** | **Study Accession** | **Genome Accession no.** | **Reads Accession no.**[1] |
| Complete genome of *S. oralis* strain A22 | | GCA_965111685 | ERR13316372<br>ERR13316380 |
| Complete genome of *S. mitis* strain B22 | | GCA_965111745 | ERR13316373<br>ERR13316381 |
| Complete genome of *S. mitis* strain C22 | | GCA_965111725 | ERR13316374<br>ERR13316382 |
| Complete genome of *S. mitis* strain D22 | PRJEB75690 | GCA_965111735 | ERR13316375<br>ERR13316383 |
| Complete genome of *S. mitis* strain E22 | | GCA_965111705 | ERR13316376<br>ERR13316384 |
| Complete genome of *S. mitis* strain F22 | | GCA_965111715 | ERR13316377<br>ERR13316385 |
| Complete genome of *S. mitis* strain G22 | | GCA_965111695 | ERR13316378<br>ERR13316386 |
| **Deposited in National Center for Biotechnology Information (NCBI) database** | | | |
| **Description** | **BioProject** | **Genome Accession no.** | **Reads Accession no.**[2] |
| Prophage OlisA1 from *S. oralis* strain A22 | | OL774868 | SRX13363554 |
| Prophage OlisA2 from *S. oralis* strain A22 | | OL774869 | SRX13395438 |
| Prophage OlisA3 from *S. oralis* strain A22 | | OL774870 | SRX13395439 |
| Prophage MissB1 from *S. mitis* strain B22 | | OL774871 | SRX13395442 |
| Prophage MissB2 from *S. mitis* strain B22 | | OL774876 | SRX13395443 |
| Prophage MissC from *S. mitis* strain C22 | | OL774872 | SRX13395444 |
| Prophage MissD from *S. mitis* strain D22 | PRJNA784746 | OL774873 | SRX13395445 |
| Prophage MissE1 from *S. mitis* strain E22 | | OL774874 | SRX13395446 |
| Prophage MissE2 from *S. mitis* strain E22 | | OL774875 | SRX13395447 |
| Prophage MissF from *S. mitis* strain F22 | | OL799250 | SRX13395448 |
| Prophage MismyG from *S. mitis* strain G22 | | OL774867 | SRX13395441 |
| Prophage MissG1 from *S. mitis* strain G22 | | OL774877 | SRX13395449 |
| Prophage MissG2 from *S. mitis* strain G22 | | OL774866 | SRX13395440 |

[1] Illumina reads are indicated first, followed by Nanopore MinION reads. [2] Illumina reads.

**Table S4. Complete genomes of *S. mitis* and *S. oralis* publicly accessible at the NCBI database at the time of this study (last accessed on August 3, 2022; as of January 8, 2024, only 1 additional complete genome of *S. mitis* has become available).**

| Species (n) | Strain | Accession number | Length in bp | Isolation source |
|---|---|---|---|---|
| *S. mitis* (10) | B6 | NC_013853.1 | 2,146,611 | Disease |
| | FDAARGOS_684 | NZ_CP046335.1 | 1,868,857 | Carriage (oral cavity) |
| | FDAARGOS_1456 | NZ_CP077259.1 | 1,868,859 | Carriage (oral cavity) |
| | KCOM 1350 | NZ_CP012646.1 | 1,906,344 | Disease |
| | NCTC12261 | NZ_CP028414.1 | 1,868,883 | Carriage (oral cavity) |
| | Nm-65 | AP023349.1 | 2,085,837 | Disease |
| | S022-V3-A4 | NZ_CP047883.1 | 2,086,958 | Carriage (saliva) |
| | S022-V7-A3 | NZ_CP067992.1 | 2,033,396 | Carriage (saliva) |
| | SK637 | NZ_CP028415.1 | 1,942,107 | Carriage (oral cavity) |
| | SVGS_061 | NZ_CP014326.1 | 2,167,922 | Disease |
| *S. oralis* (16) | 34 | NZ_CP079724.1 | 1,920,884 | Carriage (oral cavity) |
| | 1648 | NZ_CP094226.1 | 1,876,737 | Disease |
| | CCUG 53468 | NZ_CP029257.1 | 2,002,022 | Carriage (saliva) |
| | FDAARGOS_367 | NZ_CP023507.1 | 1,931,547 | Carriage (oral cavity) |
| | FDAARGOS_885 | NZ_CP065707.1 | 1,905,756 | Carriage (dental plaque) |
| | FDAARGOS_886 | NZ_CP065706.1 | 1,994,899 | Carriage (throat) |
| | FDAARGOS_1020 | NZ_CP066021.1 | 2,053,209 | Disease |
| | FDAARGOS_1021 | NZ_CP066059.1 | 2,024,323 | Disease |
| | FDAARGOS_1075 | NZ_CP066041.1 | 1,913,782 | Disease |
| | HP01 | NZ_CP097843.1 | 2,063,152 | Carriage (saliva) |
| | NCTC11427 | NZ_LR134336.1 | 1,931,548 | Carriage (oral cavity) |
| | SF100 | NZ_CP069427.1 | 1,966,910 | Disease |
| | SOD | NZ_CP046523.1 | 1,985,912 | Disease |
| | SOT | NZ_CP046524.1 | 1,938,840 | Carriage (oral cavity) |
| | S.MIT/ORALIS-351 | NZ_CP019562.1 | 1,944,085 | Carriage (oral cavity) |
| | Uo5 | NC_015291.1 | 1,958,690 | Carriage (nasal cavity) |

**Table S6A. Characteristics of the additional LPXTG proteins found among the commensal study strains.**

| Protein | Locus tag | Description | Size (aa) | Closest homologues[a] found by BLASTp[b] (n) |
|---|---|---|---|---|
| **Surf1** | SMID22_17230 | LPXTG-anchored protein | 1020 | *S. oralis* (14), *S. mitis* (11), *S. pneumoniae* (7), *S. pseudopneumoniae* (2), *S. gwangjuense* (5), *Streptococcus* sp. (13) |
| **Surf2** | SMIG22_17300 | MucBD domain-containing protein | 2929 | *S. mitis* (4), *Streptococcus* sp. (2) |
| **Surf3** | SMID22_17240 | LPXTG-anchored protein | 270 | *S. oralis* (53), *S. mitis* (16), *S. pneumoniae* (19), *S. pseudopneumoniae* (8), *Streptococcus* sp. (34) |
| **Surf4** | SMIB22_11780 | FIVAR domain-containing protein | 3055 | *S. pneumoniae* (1), *S. toyakuensis* (1). Below threshold[c]: *G. sanguinis* (9) |
| **Surf5** | SMIF22_08020 | DUF1542/FIVAR domain-containing protein | 1344 | *S. mitis* (2), *S. toyakuensis* (1), *Streptococcus* sp. (3) |
| **Surf6** | SMIC22_19480<br>SMIE22_18990<br>SMIG22_20160 | DUF1542/FIVAR domain-containing protein | 2295<br>2443<br>2887 | *S. pseudopneumoniae* (9), *S. pneumoniae* (1), *Streptococcus* sp. (5) |
| **Surf7** | SMIB22_19360<br>SMIF22_19440 | DUF1542 domain-containing protein | 3298<br>2265 | *S. mitis* (1), *Streptococcus* sp. (7) |
| **Surf8** | SMID22_15220 | DUF1542 domain-containing protein | 2981 | *S. mitis* (18), *S. oralis* (2), *S. pneumoniae* (2), *Streptococcus* sp. (2), *G. haemolysans* (2), |
| **Surf9** | SMIC22_19540 | LPXTG-anchored protein | 1388 | *S. mitis* (47), *S. pneumoniae* (6), *S. oralis* (3), *S. pseudopneumoniae* (1), *Streptococcus* sp. (23), *G. haemolysans* (2), |
| **Surf10** | SMIB22_19420<br>SMIE22_19050<br>SMIF22_19540<br>SMIG22_20220 | DUF1542 domain-containing protein | 3782<br>3778<br>3469<br>3773 | *S. mitis* (36), *S. pneumoniae* (5), *S. oralis* (3), *S. pseudopneumoniae* (1), *Streptococcus* sp. (25), *G. haemolysans* (1), |
| **Surf11**[d] | SORA22_03610<br>SORA22_08560 | Isopeptide-forming adhesin | 3500<br>2904 | *S. oralis* (162), *S. gordonii* (61), *S. mitis* (13), *Streptococcus* sp. (50) |

**Table S6A.** (*cont.*)

| Protein | Locus tag | Description | Size (aa) | Closest homologues[a] found by BLASTp[b] (n) |
|---|---|---|---|---|
| **Surf12** | SMIF22_09410 | MucBD domain-containing protein | 1482 | *S. mitis* (26), *S. pseudopneumoniae* (5), *S. oralis* (1), *S. vestibularis* (4), *Streptococcus* sp. (29), *G. haemolysans* (4), |
| **Surf13**[e] | SORA22_03620<br>SORA22_08570 | G5 domain-containing protein | 703 | *S. oralis* (123), *S. mitis* (10), *Streptococcus* sp. (31) |
| **Surf14** | SMIB22_09510<br>SMIC22_08460<br>SMIE22_09630<br>SMIF22_09860<br>SMIG22_09580 | GbpC/Spa domain-containing protein | 782<br>803<br>806<br>560<br>806 | *S. mitis* (1), *S. pneumoniae* (8), *Streptococcus* sp. (8) |
| **Surf15** | SMIF22_07440 | G5 domain-containing protein | 821 | *S. mitis* (1), *S. pseudopneumoniae* (1), *Streptococcus* sp. (8) |
| **Surf16** | SMIF22_16330 | G5 domain-containing protein | 1008 | *Streptococcus* sp. (18) |
| **Surf17** | SMIC22_07290 | G5 domain-containing protein | 943 | *S. mitis* (1), *S. pneumoniae* (3), *Streptococcus* sp. (21) |
| **Surf18** | SMIB22_07270<br>SMIE22_06710<br>SMIG22_07350 | G5 domain-containing protein | 806<br>1199<br>1299 | *S. mitis* (1), *S. pneumoniae* (2), *Streptococcus* sp. (18) |
| **Surf19** | SMIG22_04850 | PsrP fragment | 72 | *S. pneumoniae* (1088), *S. mitis* (62), *Streptococcus* sp. (43) |
| **Surf20** | SMIE22_16460<br>SMIF22_17110<br>SMIG22_17710 | SHIRT domain-containing protein | 1313<br>1108<br>1150 | *S. pneumoniae* (64), *S. mitis* (2), *Streptococcus* sp. (19) |
| **Surf21** | SMIG22_16090 | LPXTG-anchored protein | 8883 | *S. mitis* (3), *Streptococcus* sp. (3) |
| **Surf22** | SMIB22_07890 | Rib/YPDG domain-containing protein | 3834 | *S. mitis* (10), *S. pneumoniae* (3), *S. pseudopneumoniae* (2), *Streptococcus* sp. (10), *G. haemolysans* (1) |

**Table S6A.** (*cont.*)

| Protein | Locus tag | Description | Size (aa) | Closest homologues[a] found by BLASTp[b] (n) |
|---|---|---|---|---|
| **Surf23** | SMIC22_08010<br>SMIF22_08180<br>SMIG22_08020 | LPXTG-anchored protein | 3247<br>3507<br>2956 | *S. mitis* (10), *S. pneumoniae* (4), *S. oralis* (1), *Streptococcus* sp. (17), *G. haemolysans* (1) |
| **Surf24** | SMIF22_08120 | UP-LPXTG-anchored protein Surf24 | 4146 | *S. mitis* (7), *Streptococcus* sp. (9) |
| **Surf25** | SMIE22_07300 | Rib/Ig-like domain-containing protein | 5324 | *S. mitis* (11), Below threshold[f]: *S. haemolyticus* (376) |
| **Surf26** | SMID22_15240 | Rib/alpha-like domain-containing protein | 1067 | *S. mitis* (5), *Streptococcus* sp. (1) |
| **Surf27** | SORA22_19520 | LPXTG-anchored protein | 253 | *S. oralis* (78), *Streptococcus* sp. (14), *Caudoviricetes* sp. (3) |
| **Surf28** | SMID22_21850 | LPXTG-anchored protein | 941 | *S. mitis* (42), *S. pseudopneumoniae* (1), *S. gwangjuense* (1), *S. chosunense* (1), *Streptococcus* sp. (12), *Granulicatella* sp. (1) |
| **Surf29** | SMIG22_00330 | LPXTG-anchored protein | 686 | *S. pneumoniae* (4), *Streptococcus* sp. (1) |
| **Surf30** | SMIC22_00400 | LPXTG-anchored protein | 516 | *S. pneumoniae* (6), *S. mitis* (1), *Streptococcus* sp. (6) |
| **Surf31** | SMIB22_00560 | LPXTG-anchored protein | 516 | *S. mitis* (14), *S. pneumoniae* (1), *Streptococcus* sp. (6) |
| **Surf32** | SMIF22_06970 | Rib/alpha-like domain-containing protein | 2116 | *S. mitis* (30), *S. pneumoniae* (1), *S. pseudopneumoniae* (1), *Streptococcus* sp. (21), *G. haemolysans* (4), *G. elegans* (1) |
| **Surf33** | SMID22_05850 | Rib/alpha-like domain-containing protein | 1935 | *S. mitis* (9), *S. pseudopneumoniae* (1), *S. toyakuensis* (1), *G. haemolysans* (4), *G. elegans* (1) |
| **Surf34** | SMIB22_19690<br>SMIC22_19780<br>SMIE22_19290<br>SMIF22_19820<br>SMIG22_20580 | IgA-binding beta antigen | 842<br>852<br>856<br>814<br>1007 | *S. pneumoniae* (70), *S. pseudopneumoniae* (3), *S. mitis* (1), *Streptococcus* sp. (11) |

**Table S6A.** (*cont.*)

| Protein | Locus tag | Description | Size (aa) | Closest homologues[a] found by BLASTp[b] (n) |
|---|---|---|---|---|
| **Surf35** | SORA22_13860 | CshA/CshB family fibrillar adhesin-related protein | 2693 | *S. oralis* (235), *S. gordonii* (112), *S. sanguinis* (142), *S. mitis* (16), *Streptococcus* sp. (27), *Abiotrophica* sp. (3) |
| **Surf36** | SMID22_06460 | LPXTG-anchored protein | 1576 | *S. mitis* (25), *S. pseudopneumoniae* (2), *S. vestibularis* (3), *G. haemolysans* (1), *Streptococcus* sp. (4) |
| **Surf37** | SMIB22_14920<br>SMIC22_15030 | LPXTG-anchored protein | 2723<br>2619 | *S. pneumoniae* (3), *Streptococcus* sp. (24) |
| **Surf38** | SMIE22_14400<br>SMIF22_15250<br>SMIG22_15760 | LPXTG-anchored protein | 2635<br>2284<br>2634 | *S. pneumoniae* (3), *Streptococcus* sp. (17) |
| **Surf39** | SMID22_08270 | DUF1542 domain-containing protein | 2018 | *S. mitis* (12), *S. pseudopneumoniae*. (1) |
| **Surf40**[g] | SORA22_03540<br>SORA22_03560 | Antigen I/II family LPXTG-anchored adhesin | 1517<br>1541 | *S. oralis* (110), *S. gordonii* (64), *S. mutans* (183), *S. sobrinus* (34), *S. intermedius* (30), *S. anginosus* (20), *Streptococcus* sp. (53) |
| **Surf41** | SORA22_13940 | Cna B-type domain-containing protein | 1211 | *S. oralis* (152), *S. mitis* (3), *S. pseudopneumoniae* (2), *Streptococcus* sp. (40) |
| **Surf42** | SORA22_17000 | Isopeptide-forming pilin-related protein | 1051 | *S. oralis* (88), *S. mitis* (2), *Streptococcus* sp. (21), *Gemella* sp. (5) |
| **Surf43** | SORA22_03640 | LPXTG-anchored SHIRT domain periscope protein | 1356 | *S. oralis* (89), *S. gordonii* (75), *S. sanguinis* (52), *Streptococcus* sp. (21) |
| **Surf44** | SORA22_15350 | LPXTG-anchored protein | 1582 | *S. oralis* (96), *S. gordonii* (35), *S. mitis* (29), *Streptococcus* sp. (26) |
| **Surf45** | SMID22_14560 | C-terminal β-galactosidase BgaA | 195 | *S. oralis* (146), *S. mitis* (54), *S. pneumoniae* (27), *S. pseudopneumoniae* (8), *Streptococcus* sp. (53) |

**Table S6A.** (*cont.*)

| Protein | Locus tag | Description | Size (aa) | Closest homologues[a] found by BLASTp[b] (n) |
|---|---|---|---|---|
| **Surf46** | SORA22_04010<br>SMIC22_15880<br>SMID22_17860<br>SMIE22_15300<br>SMIG22_16550 | Thiol reductase thioredoxin | 345 | *S. mitis* (20), *S. oralis* (18), *S. pseudopneumoniae* (20), *S. pneumoniae* (11), *Streptococcus* sp. (26) |
| **Surf47** | SMID22_15300 | β-N-acetylglucosaminidase | 1601 | *S. oralis* (187), *S. mitis* (75), *S. pseudopneumoniae* (16), *S. infantis* (17), *S. cristatus* (9), *Streptococcus* sp. (101) |
| **Surf48** | SMID22_02990 | Alpha-L-fucosidase | 1941 | *S. oralis* (163), *S. mitis* (69), *S. pseudopneumoniae* (16), *S. infantis* (13), *Streptococcus sp.* (83) |
| **Surf49** | SMID22_19430 | β-N-acetylglucosaminidase | 2670 | *S. oralis* (181), *S. mitis* (70), *S. pseudopneumoniae* (13), *S. infantis* (14), *Streptococcus sp.* (98) |
| **Surf50** | SORA22_19300 | Cell surface ecto-5'-nucleotidase Nt5e | 727 | *S. oralis* (136), *S. gordonii* (47), *S. sanguinis* (67), *S. mitis* (14), *S. suis* (380), *S. anginosus* (42), *S. cristatus* (26), *Streptococcus* sp. (190) |
| **Sprt2** | SMID22_06800 | S8 family serine peptidase | 1573 | *S. oralis* (128), *S. mitis* (37), *S. parasanguinis* (65), *S. pseudopneumoniae* (38), *Streptococcus sp.* (82) |
| **ZmpE** | SMIB22_19470<br>SMIC22_19590<br>SMIE22_19100<br>SMIG22_20270 | Zinc metalloprotease | 1642<br>1648<br>1706<br>1607 | |
| **ZmpF** | SMIC22_05630<br>SMIE22_05200<br>SMIF22_05740 | Zinc metalloprotease | 2044 | |

[a] *S. anginosus*, *S. cristatus*, *S. gordonii*, *S. infantis*, *S. intermedius*, *S. mitis*, *S. mutans*, *S. oralis*, *S. parasanguinis*, *S. pneumoniae*, *S. pseudopneumoniae*, *S. sanguinis*, *S. sobrinus*, *S. suis, S. toyakuensis*, and *S. vestibularis* belong to the *Streptococcus* genus. *G. haemolysans* and *G. sanguinis* belong to the *Gemella* genus. *G. elegans* belong to the *Granulicatella* genus.
[b] Criteria (except when noted otherwise): query coverage >60%, amino acid identity >60%, E value <1e$^{-50}$
[b] *G. sanguinis*: query coverage >75%, amino acid identity >40%, E value <1e$^{-50}$
[d] The two Surf11 homologues from strain A shared 88% amino acid identity.
[e] The two Surf13 homologues from strain A were 100% identical.
[f] *S. haemolyticus*: query coverage >45%, amino acid identity >43%, E value <1e$^{-50}$
[g] The two Surf40 homologues from strain A shared 79% amino acid identity.

**Table S6B. Characteristics of the additional lipoproteins found among the commensal study strains.**

| Type | Protein | Locus tag | Description | Size (aa) | Lipo box | Closest homologues[a] found by BLASTp[b] (n) |
|---|---|---|---|---|---|---|
| ABC-associated | **GlnH3[c]** | SMID22_05380 | Glutamine ABC transporter substrate-binding protein | 288 | VTAC | [d]*S. pneumoniae* (412), *S. mitis* (62), *S. oralis* (37), *S. cristatus* (14), *S. gordonii* (15), *S. salivarius* (20), *S. thermophilus* (16), *Streptococcus* sp. (100) |
| | **Lipo1** | SMIF22_17450 | ABC transporter substrate-binding protein (ABC9) | 521 | LTAC | [d]*S. mitis* (27), *S. pneumoniae* (6), *S. pseudopneumoniae* (3), *S. oralis* (2), *S. parasanguinis* (15), *Streptococcus* sp. (42), Non-*Streptococcus* sp. (9) |
| | **Lipo2** | SORA22_06040<br>SMIB22_06110<br>SMID22_15470<br>SMIF22_06280 | Iron ABC transporter substrate-binding protein | 336 | MAAC | [d]*S. mitis* (127), *S. oralis* (106), *S. infantis* (15), *S. gordonii* (24), *S. sanguinis* (16), *Streptococcus* sp. (132) |
| | **Lipo3** | SMIF22_18100 | Iron ABC transporter substrate-binding protein | 346 | LSGC | [d]*S. mitis* (71), *S. oralis* (13), *S. pseudopneumoniae* (7), *S. infantis* (5) *Streptococcus* sp. (38) |
| | **Lipo4** | SMIB22_02930<br>SMIC22_02540<br>SMIE22_02630<br>SMIF22_02690<br>SMIG22_02820<br>SMID22_02260[e]<br>SMID22_02270[e] | Oligopeptide ABC transporter substrate-binding AliB-like protein | 654<br><br><br><br>99+428 | LAAC | [d]*S. mitis* (123), *S. oralis* (23), *S. pneumoniae* (6), *S. pseudopneumoniae* (10), *Streptococcus* sp. (69) |
| | **Lipo5** | SMIB22_13080<br>SMIC22_13300<br>SMID22_13600<br>SMIE22_12420<br>SMIF22_13470<br>SMIG22_13970 | Oligopeptide ABC transporter substrate-binding AliB-like protein | 655 | LAAC | [d]*S. mitis* (75), *S. pseudopneumoniae* (29), *S. pneumoniae* (12), *S. infantis* (10), *S. oralis* (1), *Streptococcus* sp. (78) |
| | **Lipo6** | SMIB22_07150<br>SMIC22_07130<br>SMIF22_07340<br>SMIG22_07220<br>SMID22_07340[f]<br>SMID22_13310[f] | Oligopeptide ABC transporter substrate-binding protein | 658<br><br><br>668 | LAAC | [d]*S. mitis* (98), *S. oralis* (21), *S. pseudopneumoniae* (7), *S. pneumoniae* (3), *Streptococcus* sp. (45) |

**Table S6B.** (*cont.*)

| Type | Protein | Locus tag | Description | Size (aa) | Lipo box | Closest homologues[a] found by BLASTp[b] (n) |
|---|---|---|---|---|---|---|
| **ABC-associated** (*cont.*) | **Lipo7** | SORA22_16930<br>SMIB22_16990<br>SMIC22_17170<br>SMIE22_16600<br>SMIF22_17260<br>SMIG22_17860 | Oligopeptide ABC transporter substrate-binding AliB-like protein | 653 | LSAC | [d]*S. mitis* (154), *S. oralis* (154), *S. pneumoniae* (81), *S. pseudopneumoniae* (13), *Streptococcus* sp. (104) |
| | **Lipo8** | SORA22_16940<br>SMIB22_17000<br>SMID22_03130<br>SMIE22_16610 | Oligopeptide ABC transporter substrate-binding AliB-like protein | 655 | LTAC | [c]*S. oralis* (123), *S. mitis* (46), *S. pneumoniae* (58), *S. infantis* (15), *S. pseudopneumoniae* (7), *Streptococcus* sp. (104) |
| | **Lipo9** | SMIF22_03840<br>SMIG22_03890 | Oligopeptide ABC transporter substrate-binding protein | 662 | LAAC | [c]*S. mitis* (56), *S. pseudopneumoniae* (9), *Streptococcus* sp. (52) |
| | **PitA$_{like}$**[g] | SORA22_17890<br>SMIB22_17890<br>SMIC22_18120<br>SMIE22_17350<br>SMIF22_18110<br>SMIG22_18540 | Iron ABC transporter substrate-binding protein | 348 | LTAC<br>LAAC | *S. pneumoniae* (199), *S. mitis* (87), *S. oralis* (23), *Streptococcus* sp. (142), Non-*Streptococcus* sp. (134) |
| **Non-ABC-associated** | **Lipo10** | SMIE22_03820 | SdpI family protein | 213 | LVGC | *S. mitis* (27), *S. pseudopneumoniae* (20), *Streptococcus* sp. (39) |
| | **Lipo11** | SMIB22_08550[h]<br>SMIE22_08060[h]<br>SMID22_10080 | EfeM/EfeO family lipoprotein | 153<br>153<br>290 | LTAC | *S. mitis* (33), *S. pseudopneumoniae* (15), *S. oralis* (35), *S. gordonii* (30), *S. sanguinis* (51), *S. parasanguinis* (37), *S. cristatus* (17), *S. salivarius* (38), *S. thermophilus* (33), *Streptococcus* sp. (180), *Gemella* sp. (13), *Glaesserella* sp. (29), Other non-*Streptococcus* sp. (115) |
| | **Lipo12** | SMIB22_00800<br>SMID22_10070<br>SMIE22_00650 | DUF6287 domain-containing protein | 109<br>348<br>348 | LTAC | *S. mitis* (29), *S. pseudopneumoniae* (12), *Streptococcus sp.* (21) |
| | **Lipo13** | SORA22_19410<br>SMID22_21720 | Hypothetical protein | 227<br>232 | LGAC | *S. oralis* (95), *S. mitis* (108), *S. pseudopneumoniae* (20), *S. infantis* (12), *Streptococcus* sp. (122) |

**Table S6B.** (*cont.*)

| Type | Protein | Locus tag | Description | Size (aa) | Lipo box | Closest homologues[a] found by BLASTp[b] (n) |
|---|---|---|---|---|---|---|
| **Non-ABC-associated** (*cont.*) | **Lipo14** | SMIB22_01180<br>SMIC22_00930<br>SMID22_00810<br>SMIE22_01010<br>SMIF22_00850<br>SMIG22_00840 | Carbohydrate-binding domain-containing protein | 410<br>403<br>405<br>403<br>410<br>412 | MTAC | *S. mitis* (50), *S. oralis* (132), *S. infantis* (17), *S. parasanguinis* (59), *S. salivarius* (89), *Streptococcus* sp. (183) |
| | **Lipo15** | SMID22_16160 | Hypothetical protein | 326 | LVGC | *S. oralis* (42), *S. mitis* (4), *S. gordonii* (15), *Streptococcus* sp, (20) |
| | **Lipo16** | SORA22_05570 | LemA family protein | 198 | LGSC | *S. oralis* (30), *S. mitis* (3), *S. infantis* (11), *Streptococcus* sp. (34) |
| | **Lipo17** | SORA22_11740 | Hypothetical protein | 209 | LASC | *S. oralis* (93), *S. mitis* (17), *Streptococcus* sp. (52) |
| | **Lipo18** | SORA22_11040 | DUF4352 domain-containing protein | 384 | LVAC | *S. oralis* (52), *S. mitis* (9), *Streptococcus* sp. (36) |
| | **Lipo19** | SORA22_07770 | Hypothetical protein | 222 | LNGC | *S. oralis* (59), *S. mitis* (26), *S, infantis* (15), *S, gordonii* (45), *S. sanguinis* (29), *Streptococcus* sp. (47) |
| | **Lipo20** | SORA22_07050 | Polysaccharide deacetylase family protein | 463 | LLGC | *S. oralis* (136), *S. mitis* (128), *S. pneumoniae* (584), *S. pseudopneumoniae* (30), *Streptococcus* sp. (149) |
| | **Lipo21** | SORA22_14990 | DUF1307 domain-containing protein | 183 | LLGC | *S. oralis* (132), *S. mitis* (5), *S. pseudopneumoniae* (4), *Streptococcus* sp. (51) |
| | **Lipo22** | SORA22_15000 | DUF1307 domain-containing protein | 183 | LLGC | *S. oralis* (113), *S. mitis* (5), *S. pseudopneumoniae* (4), *S. infantis* (5), *Streptococcus* sp. (44) |
| | **Lipo23** | SORA22_17650 | 5'-nucleotidase lipoprotein e(P4) family | 285 | LTGC | *S. oralis* (52), *S. mitis* (33), *S. parasanguinis* (25), *S. gordonii* (14), *S. anginosus* (28), *S. dysgalactiae* (22), *S. equi* (17), *S. pyogenes* (49), *Streptococcus* sp. (139), *Gemella* sp. (13) |
| | **Lipo24** | SORA22_15380<br>SMID22_04160 | Hypothetical protein | 254 | LASC | *S. oralis* (23), *S. mitis* (4), *S. infantis* (12), *Streptococcus* sp. (20) |

**Table S6B.** (*cont.*)

| Type | Protein | Locus tag | Description | Size (aa) | Lipo box | Closest homologues[a] found by BLASTp[b] (n) |
|---|---|---|---|---|---|---|
| **Non-ABC-associated** (*cont.*) | **Lipo25**[i] | SORA22_00360<br>MissE22_0004<br>SMIG22_10600 | 3-dehydroquinate synthase | 107<br>105<br>106 | LVAC | *Caudoviricetes* sp. (76), *S. oralis* (21), *S. mitis* (11), *S. pneumoniae* (134) |
| | **Lipo26** | SMIB22_09260<br>SMIF22_09630<br>SMIG22_09330 | D-ribose-binding protein | 321 | LVAC | *S. mitis* (10), *S. pneumoniae* (10), *S. uberis* (24), *S. dysgalactiae* (27), *S. agalactiae* (54), *Streptococcus* sp. (89), *Lactococcus* sp. (55) |
| | **Lipo27** | SMIB22_00810<br>SMID22_10050 | Hypothetical protein | 304 | LTAC | *S. mitis* (3), *Streptococcus* sp. (4) |
| | **Lipo28** | SMID22_01020[j]<br>SMID22_06220[j] | Hypothetical protein | 173 | LTAC | *S. mitis* (2), *S. oralis* (4), *Streptococcus* sp. (5) |
| | **Lipo29** | SORA22_07350 | Hypothetical protein | 319 | LGAC | *S. oralis* (37), *S. mitis* (14), *Streptococcus* sp. (20), *Caudoviricetes* sp. (3) |
| | **Lipo30** | SMIC22_09280 | DUF6287 domain-containing protein | 186 | LTAC | *S. mitis* (41), *S. pseudopneumoniae* (16), *Streptococcus* sp. (32) |
| | **Lipo31** | SMID22_04450 | Hypothetical protein | 173 | LAAC | *S. mitis* (6), *S. oralis* (27), *S. sanguinis* (13), *S. gordonii* (13), *Streptococcus* sp. (32) |
| | **Lipo32** | SMID22_06010 | DUF1310 family protein | 131 | VGGC | *S. oralis* (28), *S. mitis* (18), *S. intermedius* (7), *S. suis* (7), *Streptococcus* sp. (28) |
| | **Lipo33** | SMID22_06030 | DUF1310 family protein | 132 | VGGC | *S. oralis* (31), *S. mitis* (19), *S. intermedius* (15), *S. sanguinis* (14), *Streptococcus* sp. (35) |
| | **Lipo34** | SMID22_06050 | DUF1310 family protein | 134 | IGGC | *S. oralis* (32), *S. mitis* (19), *S. suis* (16), *S. sanguinis* (17), *S. intermedius* (8), *Streptococcus* sp. (38) |

**Table S6B.** (*cont.*)

| Type | Protein | Locus tag | Description | Size (aa) | Lipo box | Closest homologues[a] found by BLASTp[b] (n) |
|---|---|---|---|---|---|---|
| **Non-ABC-associated** (*cont.*) | **Lipo35** | SMID22_17180[k]<br>SMID22_17190[k] | Lipoprotein | 164<br>165 | LGAC | *S. oralis* (22), *S. mitis* (7), *S. pneumoniae* (12), *Streptococcus* sp. (22) |
| | **Lipo36** | SMIG22_08400 | Lipoprotein | 51 | LSAC | *S. mitis* (19), *Streptococcus* sp. (16) |
| | **Lipo37**[l] | SMIG22_10830 | Lipoprotein | 148 | LVAC | *Caudoviricetes* sp. (19), *S. mitis* (10), *Streptococcus* sp. (13) |
| | **$NisI_{like}$** | SMID22_12280 | NisI/SpaI family lantibiotic immunity lipoprotein | 220 | LTGC | *S. mitis* (18), *Streptococcus* sp. (9) |

[a] *S. agalactiae*, *S. anginosus*, *S. cristatus*, *S. dysgalactiae*, *S. equi*, S. *infantis, S. intermedius*, *S. gordonii*, S. *mitis*, *S. oralis*, *S. parasanguinis*, S. *pneumoniae*, *S. pseudopneumoniae*, *S. pyogenes*, S. *salivarius*, *S. sanguinis*, *S.* suis, S. *thermophilus*, and *S. uberis*, belong to the *Streptococcus* genus.
[b] Criteria (except when noted otherwise): query coverage >60%, amino acid identity >60%, E value $<1e^{-50}$
[c] GlnH3 is homologous to the substrate-binding protein of one of the six pneumococcal glutamine uptake transporters (GlnHQP) (1). However, *glnH3* is a pseudogene in most pneumococcal strains.
[d] Due to high sequence similarity between peptide ABC transporters, the closest homologues were selected based on 80% (instead of 60%) amino acid identity.
[e] Lipo4 in strain D22 is divided into two open reading frames, one encoding the first 99 amino acids and a second encoding an initial methionine and the last 428 amino acids.
[f] The two Lipo6 homologues from strain D22 were 98.8% identical.
[g] PitA-like is homologous to the substrate-binding protein of one of the three pneumococcal iron uptake transporters (PitABCD) (2). However, pneumococcal *pitA* encodes an iron-binding non-lipoprotein as it lacks the lipobox motif.
[h] The Lipo11 homologues from strains B22 and E22 were truncated in the C-terminus.
[i] Lipo25 was encoded in prophages OlisA1 in strain A22 (107 aa), MissE2 in strain E22 (105 aa), and MismyG in strain G22 (106 aa)
[j] The two Lipo28 homologues from strain D22 were 100% identical.
[k] The two Lipo35 homologues from strain D22 shared 85.0% amino acid identity.
[l] Lipo37 was encoded in prophage MismyG in strain G22.

**References**


1. Hoskins J, Alborn Jr WE, Arnold J, Blaszczak LC, Burgett S, DeHoff BS, Estrem ST, Fritz L, Fu D-J, Fuller W. 2001. Genome of the bacterium *Streptococcus pneumoniae* strain R6. J Bacteriol 183:5709-5717.

2. Brown JS, Gilliland SM, Ruiz-Albert J, Holden DW. 2002. Characterization of Pit, a *Streptococcus pneumoniae* iron uptake ABC transporter. Infect Immun 70:4389-98.

**Table S6C. Characteristics of the novel choline-binding proteins found among the commensal study strains.**

| Protein | Locus tag | Description | Size (aa) | Closest homologues[a] found by BLASTp[b] (n) |
|---|---|---|---|---|
| **Cbp20** | SORA22_14600<br>SMIB22_08540<br>SMIC22_11430<br>SMID22_05160<br>SMIE22_08050<br>SMIF22_08790<br>SMIG22_08640 | Cell wall-associated N-acetylmuramoyl-L-alanine amidase | 600<br>465<br>465<br>565<br>465<br>465<br>465 | *S. mitis* (113), *S. pneumoniae* (11), *S. pseudopneumoniae* (12), *S. oralis* (163), *S. infantis* (22), *Streptococcus* sp. (108) |
| **Cbp21** | SMIE22_08790<br>SMIG22_11140 | Cell wall-associated murein hydrolase | 288<br>288 | *Caudoviricetes* sp. (79), *S. mitis* (12), *S. oralis* (3), *S. pneumoniae* (8), *S. pseudopneumoniae* (1), *S. infantis* (1), *S. toyakuensis* (1), *Streptococcus* sp. (8) |
| **Cbp22** | SMIG22_00300 | Choline-binding protein | 303 | *S. pneumoniae* (1), *S. mitis* (2), *Streptococcus* sp. (1) |
| **Cbp23** | SMIB22_00530 | Choline-binding protein | 316 | *S. mitis* (3), *S. pneumoniae* (88), *Streptococcus* sp. (2), *L. monocytogenes* (1) |
| **Cbp24** | SMIC22_00360 | Choline-binding protein | 342 | - |
| **Cbp25** | SMIC22_09260 | CAP domain-containing protein | 457 | *S. pseudopneumoniae* (3), *S. mitis* (1) |
| **Cbp26** | SMIE22_00360 | Choline-binding protein | 462 | *S. mitis* (1), *Streptococcus* sp. (1) |
| **Cbp27** | SMIF22_00310 | Choline-binding protein | 491 | *S. mitis* (1), *S. pseudopneumoniae*. (1) |
| **Cbp28** | SMIC22_00370 | Choline-binding protein | 200 | - |
| **Cbp29** | SMIB22_00540 | Choline-binding protein | 326 | *S. mitis* (14), *S. pneumoniae* (1), *Streptococcus* sp. (7), *L. monocytogenes* (1) |
| **Cbp30** | SMIB22_00550 | Choline-binding protein | 456 | *S. mitis* (64), *S. pneumoniae* (16), *S. pseudopneumoniae* (3), *S. toyakuensis* (3), *Streptococcus* sp. (14), *L. monocytogenes* (2) |
| **Cbp31** | SMIG22_00310 | Choline-binding protein | 494 | *S. mitis* (19), *S. pneumoniae* (2), *S. pseudopneumoniae* (1), *Streptococcus sp.* (12) |
| **Cbp32** | SMIC22_00380 | Choline-binding protein | 517 | *S. mitis* (34), *S. pneumoniae* (3), *S. pseudopneumoniae* (2), *Streptococcus sp.* (13) |

**Table S6C.** (*cont.*)

| Protein | Locus tag | Description | Size (aa) | Closest homologues[a] found by BLASTp[b] (n) |
|---|---|---|---|---|
| **Cbp33** | SMIC22_05310 | Neuraminidase-like protein | 1188 | *S. pseudopneumoniae* (45), *S. pneumoniae* (31), *S. mitis* (33), *S. oralis* (1), *Streptococcus* sp. (34), *G. haemolysans* (1) |
| **Cbp34** | SMIC22_17460 | SEC10/PgrA surface exclusion domain-containing protein | 1017 | *S. mitis* (5), *S. pseudopneumoniae* (1), *S. pneumoniae* (1), *Streptococcus sp.*(4) |
| **Cbp35** | SMIC22_00900<br>SMIE22_00980 | Choline-binding PspA-like protein | 748<br>692 | *S. pneumoniae* (817), *Streptococcus* sp. (4) |
| **Cbp36** | SMIB22_01150 | Choline-binding protein | 985 | *S. vestibularis*[c] (9), *S. mitis*[c] (55), *S. pseudopneumoniae*[c] (2), *S. oralis*[c] (1), *Streptococcus* sp.[c] (18), *S. pneumoniae* (39), *G. haemolysans* (5) |

[a] *S. infantis, S. mitis*, *S. oralis*, *S. pneumoniae*, *S. pseudopneumoniae*, *S. toyakuensis,* and *S. vestibularis* belong to the *Streptococcus* genus. *G. haemolysans* belongs to the *Gemella* genus. *L. monocytogenes* belongs to the *Listeria* genus.
[b] Criteria: query coverage >60%, amino acid identity >60%, E value <$1e^{-50}$
[c] LPXTG proteins

**Table S7A. Characteristics of the restriction-modification (RM) systems found among the commensal study strains.**

| Type | System | Strain | Locus tag | Size (aa) | Function[a] | Description | Closest homologues[b] found by BLASTp[c] |
|---|---|---|---|---|---|---|---|
| I | I1 | B22 | SMIB22_01390 | 1120 | REase | Type I RM system subunit HsdR | *S. pseudopneumoniae, S. mitis*, *S. suis*, *Streptococcus* sp., *Enterococcus* sp., *Lactococcus* sp., *Bifidobacterium* sp., *Listeria* sp., Non-*Streptococcus* sp. |
| | | | SMIB22_01400 | 356 | Other | Phage abortive infection protein | |
| | | | SMIB22_01410 | 365 | Specificity | Type I RM system subunit HsdS | |
| | | | SMIB22_01420 | 533 | MTase | Type I RM system subunit HsdM | |
| | I2.1 | C22 | SMIC22_01160 | 1120 | REase | Type I RM system subunit HsdR | *S. pseudopneumoniae*, *S. mitis*, *S. suis*, *Streptococcus* sp., *Enterococcus* sp., *Staphylococcus* sp., Non-*Streptococcus* sp. |
| | | | SMIC22_01170 | 413 | Other | Fic family protein | |
| | | | SMIC22_01180 | 351 | Other | Phage abortive infection protein | |
| | | | SMIC22_01190 | 394 | Specificity | Type I RM system subunit HsdS | |
| | | | SMIC22_01200 | 533 | MTase | Type I RM system subunit HsdM | |
| | | G22 | SMIG22_01210 | 1120 | REase | Type I RM system subunit HsdR | |
| | | | SMIG22_01220 | 413 | Other | Fic family protein | |
| | | | SMIG22_01230 | 351 | Other | Phage abortive infection protein | |
| | | | SMIG22_01240 | 394 | Specificity | Type I RM system subunit HsdS | |
| | | | SMIG22_01250 | 533 | MTase | Type I RM system subunit HsdM | |
| | I2.2 | E22 | SMIE22_01230 | 1120 | REase | Type I RM system subunit HsdR | *S. pseudopneumoniae*, *S. mitis*, *S. suis*, *Streptococcus* sp., *Enterococcus* sp., *Lactococcus* sp., *Lacticaseibacillus* sp., *Lactobacillus* sp., Non-*Streptococcus* sp. |
| | | | SMIE22_01240 | 413 | Other | Fic family protein | |
| | | | SMIE22_01250 | 355 | Other | Phage abortive infection protein | |
| | | | SMIE22_01260 | 369 | Specificity | Type I RM system subunit HsdS | |
| | | | SMIE22_01270 | 490 | MTase | Type I RM system subunit HsdM | |
| | I2.3 | F22 | SMIF22_01010[d] SMIF22_01020[d] | 68+1021 | REase | Type I RM system subunit HsdR | *S. pseudopneumoniae*, *S. mitis*, *S. suis*, *S. pyogenes*, *Streptococcus* sp., *Enterococcus* sp., Non-*Streptococcus* sp. |
| | | | SMIF22_01030 | 413 | Other | Fic family protein | |
| | | | SMIF22_01040 | 494 | Other | Phage abortive infection protein | |
| | | | SMIF22_01050 | 396 | Specificity | Type I RM system subunit HsdS | |
| | | | SMIF22_01060 | 533 | MTase | Type I RM system subunit HsdM | |
| | I3 | D22 | SMID22_03790 | 996 | REase | Type I RM system subunit HsdR | *S. mitis*, *S. oralis*, *S. gordonii*, *S. parasanguinis*, *S. salivarius*, *S. thermophilus*, *Streptococcus* sp. |
| | | | SMID22_03800 | 71 | Other | Hypothetical protein | |
| | | | SMID22_03810 | 108 | Other | Hypothetical protein | |
| | | | SMID22_03820 | 99 | Other | Hypothetical protein | |
| | | | SMID22_03830 | 269 | Other | Hypothetical protein | |
| | | | SMID22_03840 | 401 | Specificity | Type I RM system subunit HsdS | |
| | | | SMID22_03850 | 179 | Other | Hypothetical protein | |
| | | | SMID22_03860 | 273 | Other | Hypothetical protein | |
| | | | SMID22_03870 | 533 | MTase | Type I RM system subunit HdsM | |

**Table S7A.** (*cont.*)

| Type | System | Strain | Locus tag | Size (aa) | Function[a] | Description | Closest homologues[b] found by BLASTp[c] |
|---|---|---|---|---|---|---|---|
| I (*cont.*) | I4.1 | C22 | SMIC22_12930 | 1116 | REase | Type I RM system subunit HsdR | *S. pneumoniae*, *S. pseudopneumoniae*, *S. oralis*, *S. mitis*, *Streptococcus* sp. |
| | | | SMIC22_12940 | 355 | Specificity | Type I RM system subunit HsdS | |
| | | | SMIC22_12950 | 321 | Other | Tyrosine recombinase | |
| | | | SMIC22_12960 | 137 | Other | Type II toxin Phd | |
| | | | SMIC22_12970 | 78 | Other | Type II antitoxin Doc | |
| | | | SMIC22_12980 | 373 | Specificity | Type I RM system subunit HsdS | |
| | | | SMIC22_12990 | 497 | MTase | Type I RM system subunit HsdM | |
| | I4.2 | G22 | SMIG22_13610 | 1116 | REase | Type I RM system subunit HsdR | |
| | | | SMIG22_13620 | 193 | Specificity | Type I RM system subunit HsdS | |
| | | | SMIG22_13630 | 321 | Other | Tyrosine recombinase | |
| | | | SMIG22_13640 | 137 | Other | Type II toxin Phd | |
| | | | SMIG22_13650 | 78 | Other | Type II antitoxin Doc | |
| | | | SMIG22_13660 | 380 | Specificity | Type I RM system subunit HsdS | |
| | | | SMIG22_13670 | 180 | Specificity | Type I RM system subunit HsdS | |
| | | | SMIG22_13680 | 497 | MTase | Type I RM system subunit HsdM | |
| | I4.3 | F22 | SMIF22_13070 | 1116 | REase | Type I RM system subunit HsdR | |
| | | | SMIF22_13080 | 376 | Specificity | Type I RM system subunit HsdS | |
| | | | SMIF22_13090 | 197 | Specificity | Type I RM system subunit HsdS | |
| | | | SMIF22_13100 | 321 | Other | Tyrosine recombinase | |
| | | | SMIF22_13110 | 137 | Other | Type II toxin Phd | |
| | | | SMIF22_13120 | 78 | Other | Type II antitoxin Doc | |
| | | | SMIF22_13130 | 380 | Specificity | Type I RM system subunit HsdS | |
| | | | SMIF22_13140 | 166 | Specificity | Type I RM system subunit HsdS | |
| | | | SMIF22_13150 | 197 | Specificity | Type I RM system subunit HsdS | |
| | | | SMIF22_13160 | 180 | Specificity | Type I RM system subunit HsdS | |
| | | | SMIF22_13170 | 497 | MTase | Type I RM system subunit HsdM | |
| **II** | BcgI-like | C22 | SMIC22_15630 | 179 | REase | REase BcgI specificity subunit beta | *S. mutans, S. oralis, Streptococcus* sp. |
| | | | SMIC22_15640 | 366 | REase | REase BcgI specificity subunit beta | |
| | | | SMIC22_15650 | 336 | Other | RhuM family protein | |
| | | | SMIC22_15660 | 660 | RE-MTase | DNA adenine MTase BcgI subunit alpha | |

**Table S7A.** (*cont.*)

| Type | System | Strain | Locus tag | Size (aa) | Function[a] | Description | Closest homologues[b] found by BLASTp[c] |
|---|---|---|---|---|---|---|---|
| **II** (*cont.*) | DpnI-like | A22 | SORA22_04900 | 254 | REase[e] | Type II REase DpnC | *S. pneumoniae*, *S. oralis, Streptococcus* sp. |
| | | | SORA22_04910[d] SORA22_04920[d] | 98+55 | MTase[e] | Type II MTase DpnD | |
| | | F22 | SMIF22_04160 | 254 | REase[e] | Type II REase DpnC | |
| | | | SMIF22_04170[d] SMIF22_04180[d] | 98+55 | MTase[e] | Type II MTase DpnD | |
| | DpnII-like | D22 | SMIC22_16520 | 302 | REase[e] | Type II REase DpnB | *S. pneumoniae*, *S. mitis*, *S. oralis*, *Streptococcus* sp. |
| | | | SMID22_16530 | 190 | Other | Hypothetical protein | |
| | | | SMID22_16540 | 255 | MTase[e] | DNA cytosine MTase DpnA | |
| | | | SMID22_16550 | 279 | MTase[e] | DNA adenine MTase DpnM | |
| | EcoRI-like | B22 | SMIB22_19630 | 279 | REase | EcoRI family type II REase | *S. pseudopneumoniae, S. suis* |
| | | | SMIB22_19640 | 328 | MTase | EcoRI family adenine-specific MTase | |
| | EcoRV-like | F22 | SMIF22_07900 | 276 | REase[f] | EcoRV family type II REase | *Streptococcus* sp., Non-*Streptococcus* sp. |
| | | | SMIF22_07910 | 304 | MTase[f] | Dam family N6-adenine-specific MTase | |
| | | G22 | SMIG22_07840 | 276 | REase[f] | EcoRV family type II REase | |
| | | | SMIG22_07850 | 304 | MTase[f] | Dam family N6-adenine-specific MTase | |
| | II1 | C22 | SMIC22_07830 | 272 | REase[g] | ApaLI family REase | *S. pneumoniae*, Non-*Streptococcus* sp. |
| | | | SMIC22_07820 | 396 | MTase[g] | DNA modification MTase M.XbaI | |
| | II2 | B22 | SMIB22_15450 | 525 | REase | Type II REase | *S. mitis*, *S. oralis*, *S. gordonii*, *S. parasanguinis* |
| | | | SMIB22_15460 | 406 | MTase | DNA cytosine MTase | |
| | | D22 | SMID22_17550 | 525 | REase | Type II REase | |
| | | | SMID22_17560 | 406 | MTase | DNA cytosine MTase | |
| | II3 | F22 | SMIF22_13530 | 272 | REase[h] | Eco47II family type II REase | *Streptococcus* sp., *Clostridiales* sp., *Ruminococcus* sp., *Lachnospiraceae* sp. |
| | | | SMIF22_13540 | 333 | MTase[h] | DNA cytosine MTase | |
| | II4 | D22 | SMID22_07700 | 351 | MTase | DNA cytosine MTase | *S. pneumoniae*, *S. mitis, S. mutans*[d] |
| | | | SMID22_07710 | 466 | REase[i] | Type II REase BpuJI N-terminal | |
| | | | SMID22_07720 | 346 | MTase[i] | DNA C5-cytosine-specific MTase | |
| | | | SMID22_07730 | 264 | Other | Abortive infection family protein | |
| | | | SMID22_07740 | 187 | Other | SLATT domain-containing protein | |

**Table S7A.** (*cont.*)

| Type | System | Strain | Locus tag | Size (aa) | Function[a] | Description | Closest homologues[b] found by BLASTp[c] |
|---|---|---|---|---|---|---|---|
| II (*cont.*) | IIG1 | A22 | SORA22_15640<br>SORA22_15650 | 1461<br>224 | RE-MTase<br>MTase | Eco57I MTase domain-cont. protein<br>DNA adenine MTase | *S. suis*, *S. infantarius*, *S. thermophilus*, *Streptococcus* sp., *Lactococcus* sp. |
| | IIG2 | B22 | SMIB22_09400<br>SMIB22_09410<br>SMIB22_09420 | 632<br>316<br>1084 | MTase<br>Other<br>REase | Eco57I MTase domain-cont. protein<br>Abortive infection family protein<br>Type II REase/helicase family protein | *S. pseudopneumoniae*, *S. pneumoniae*, *S. mitis*, *S. oralis*, *S. suis*, *Streptococcus* sp. |
| | | C22 | SMIC22_08350<br>SMIC22_08360<br>SMIC22_08370 | 632<br>316<br>1085 | MTase<br>Other<br>REase | Eco57I MTase domain-cont. protein<br>Abortive infection family protein<br>Type II REase/helicase family protein | |
| | | E22 | SMIE22_09520<br>SMIE22_09530<br>SMIE22_09540 | 623<br>316<br>1085 | MTase<br>Other<br>REase | Eco57I MTase domain-cont. protein<br>Abortive infection family protein<br>Type II REase/helicase family protein | |
| | | G22 | SMIG22_09470<br>SMIG22_09480<br>SMIG22_09490 | 627<br>316<br>1085 | MTase<br>Other<br>REase | Eco57I MTase domain-cont. protein<br>Abortive infection family protein<br>Type II REase/helicase family protein | |
| IV | IV1 | B22 | SMIB22_17400<br>SMIB22_17410 | 348<br>718 | M-dep REase | 5-methylcytosine-specific REase McrC<br>AAA+ family ATPase McrB family protein | *S. suis*, *S. oralis*, Non-*Streptococcus* sp. |
| | IV2 | C22 | SMIC22_10840<br>SMIC22_10850 | 434<br>561 | M-dep REase | 5-methylcytosine-specific REase McrC<br>AAA+ family ATPase McrB family protein | *S. pneumoniae, S. mitis*, *S. oralis*, *Streptococcus* sp. |
| | IV3 | D22 | SMID22_12970<br>SMID22_12980 | 447<br>577 | M-dep REase | 5-methylcytosine-specific REase McrC<br>AAA+ family ATPase McrB family protein | |
| | IV4 | F22 | SMIF22_09380<br>SMIF22_09370 | 438<br>570 | M-dep REase | 5-methylcytosine-specific REase McrC<br>AAA+ family ATPase McrB family protein | |

[a] REase – restriction endonuclease; MTase – methyltransferase; RE-MTase – Restriction endonuclease and methyltransferase; M-dep REase – modification-dependent restriction endonuclease; domain-cont. – domain-containing

[b] *S. cristatus*, *S. equi*, *S. gordonii*, *S. infantarius*, *S. mitis*, *S. mutans*, *S. oralis*, *S. parasanguinis*, *S. pneumoniae*, *S. pseudopneumoniae*, *S. pyogenes*, *S. sanguinis*, *S. suis*, and *S. thermophilus* belong to the *Streptococcus* genus.

[c] Criteria: query coverage >60%, amino acid identity >60%, E value <$1e^{-15}$; no homologues were searched for proteins whose function was designated as “other”.

[d] REase from RM I2.3 and MTase from RM DpnI-like are each divided into two open reading frames.

[e] Predicted recognition sequence for RM DpnI-like and DpnII-like: 5’-GATC-3’

[f] Predicted recognition sequence for RM EcoRV-like: 5’-GATATC-3’
[g] Predicted recognition sequence for RM II1: 5’-TCTAGA-3’
[h] Predicted recognition sequence for RM II3: 5’-GGNCC-3’
[i] Predicted recognition sequence for RM II4: 5’-CCCGC-3’

**Table S7B. Characteristics of the type II toxin-antitoxin (TA) systems found among the commensal study strains.**

| System | Strain | Locus tag | Size (aa) | Description | Closest homologues[a] found by BLASTp[b] |
|---|---|---|---|---|---|
| COG2856CA-Xre | B22, C22, F22, G22 | SMIB22_03430<br>SMIC22_02990<br>SMIF22_03230<br>SMIG22_03330 | 182<br>177<br>177<br>182 | Type II toxin COG2856C | *S. pneumoniae*, *S. pseudopneumoniae* |
| | | SMIB22_03420<br>SMIC22_02980<br>SMIF22_03220<br>SMIG22_03320 | 252 | Type II toxin COG2856A | |
| | | SMIB22_03410<br>SMIC22_02970<br>SMIF22_03210<br>SMIG22_03310 | 112 | Type II antitoxin Cre | |
| HicAB | F22 | SMIF22_04600 | 59 | Type II toxin HicA | *S. pneumoniae*, *S. mitis*, *S. suis*, *Streptococcus* sp. |
| | | SMIF22_04610 | 150 | Type II antitoxin HicB | |
| HigBA | D22 | SMID22_03940 | 121 | Type II toxin HigB | *S. oralis*, *S. mitis*, *S. parasanguinis*, *S. suis*, *Streptococcus* sp. |
| | | SMID22_03950 | 97 | Type II antitoxin HigA | |
| MazFE | A22, B22, C22, F22, G22 | SORA22_10660<br>SMIB22_10800<br>SMIC22_20660<br>SMIF22_11080<br>SMIG22_11460 | 118<br>117<br>117<br>117<br>117 | Type II toxin MazF | *S. oralis*, *S. pseudopneumoniae*, *Streptococcus* sp. |
| | | SORA22_10670<br>SMIB22_10790<br>SMIC22_20670<br>SMIF22_11070<br>SMIG22_11450 | 71 | Type II antitoxin MazE | |
| MazFE-like | E22 | SMIE22_00390 | 117 | Type II toxin MazF-like | *S. mitis*, Non-*Streptococcus* sp. |
| | | SMIE22_00380 | 100 | Type II antitoxin MazE-like | |

**Table S7B.** (*cont.*)

| System | Strain | Locus tag | Size (aa) | Description | Closest homologues[a] found by BLASTp[b] |
|---|---|---|---|---|---|
| ParE-RelB-like1 | B22, C22, D22, E22, F22, G22 | SMIB22_01360<br>SMIC22_01120<br>SMID22_01080<br>SMIE22_01200<br>SMIF22_00980<br>SMIG22_01170 | 115 | Type II toxin ParE-like1 | *S. mitis*, *S. pseudopneumoniae*, *S. equi*, *S. suis*, *Streptococcus* sp. |
| | | SMIB22_01350<br>SMIC22_01110<br>SMID22_01070<br>SMIE22_01190<br>SMIF22_00970<br>SMIG22_01160 | 95 | Type II antitoxin RelB-like1 | |
| ParE-RelB-like2 | B22, C22, F22, G22 | SMIB22_08570<br>SMIC22_11420<br>SMIF22_08800<br>SMIG22_08660 | 115<br>115<br>101<br>117 | Type II toxin ParE-like2 | *S. mitis*, *S. oralis*, *Streptococcus* sp |
| | | SMIB22_08580<br>SMIC22_11410<br>SMIF22_08810<br>SMIG22_08670 | 94 | Type II antitoxin RelB-like2 | |
| ParE-RelB-like3 | B22, E22, F22 | SMIB22_14910<br>SMIE22_14370<br>SMIF22_15240 | 113 | Type II toxin ParE-like3 | *S. pneumoniae*, *Streptococcus* sp |
| | | SMIB22_14900<br>SMIE22_14360<br>SMIF22_15230 | 95 | Type II antitoxin RelB-like3 | |

**Table S7B.** (*cont.*)

| System | Strain | Locus tag | Size (aa) | Description | Closest homologues[a] found by BLASTp[b] |
|---|---|---|---|---|---|
| PezTA | B22, C22, D22, F22, G22 | SMIB22_17360<br>SMIC22_09890<br>SMID22_12170<br>SMIF22_17500<br>SMIG22_12250 | 253<br>253<br>253<br>253<br>256 | Type II toxin PezT | *S. mitis*, *S.* oralis, S. *parasanguinis*, *S. mutans*, *Streptococcus* sp. |
| | | SMIB22_17370<br>SMIC22_09900<br>SMID22_12180<br>SMIF22_17510<br>SMIG22_12260 | 158<br>158<br>158<br>290<br>158 | Type II antitoxin PezA | |
| Phd-Doc | C22, F22, G22 | SMIC22_12960<br>SMIF22_13110<br>SMIG22_13640 | 137 | Type II toxin Phd | *S. pneumoniae*, *S. oralis*, *S. mitis*, *S. sanguinis*, *Streptococcus* sp. |
| | | SMIC22_12970<br>SMIF22_13120<br>SMIG22_13650 | 78 | Type II antitoxin Doc | |
| RelE1B1 | B22, C22, G22 | SMIB22_17630<br>SMIC22_17750<br>SMIG22_18260 | 92 | Type II toxin RelE1 | *S. pneumoniae*, *S. mitis*, *S. sanguinis*, *S. parasanguinis*, *Streptococcus* sp. |
| | | SMIB22_17640<br>SMIC22_17760<br>SMIG22_18270 | 87 | Type II antitoxin RelB1 | |
| RelE2B2 | B22, C22, D22, E22, G22 | SMIB22_09390<br>SMIC22_08340<br>SMID22_11730<br>SMIE22_09510<br>SMIG22_09460 | 87 | Type II toxin RelE2 | *S. pneumoniae*, *S. mitis*, *S.pseudopneumoniae, Streptococcus* sp. |
| | | SMIB22_09380<br>SMIC22_08330<br>SMID22_11740<br>SMIE22_09500<br>SMIG22_09450 | 80 | Type II antitoxin RelB2 | |

**Table S7B.** (*cont.*)

| System | Strain | Locus tag | Size (aa) | Description | Closest homologues[a] found by BLASTp[b] |
|---|---|---|---|---|---|
| RelE-HigA | D22 | SMID22_03900 | 112 | Type II toxin RelE | *S. gordonii*, *S. sanguinis*, *S. cristatus*, *S. oralis*, *S. salivarius, Streptococcus* sp. |
| | | SMID22_03910 | 361 | Type II antitoxin HigA | |
| RnlAB | G22 | SMIG22_00710 | 395 | Type II toxin RnlA | Non-*Streptococcus* sp. |
| | | SMIG22_00720 | 120 | Type II antitoxin RnlB | |
| YoeB-YefM | B22, C22, F22 | SMIB22_04970<br>SMIC22_04740<br>SMIF22_04940 | 84 | Type II toxin YoeB | *S. oralis*, *S. mitis*, *S. pneumoniae*, *S. pseudopneumoniae*, *S. salivarius, S. parasanguinis*, Streptococcus sp. |
| | | SMIB22_04960<br>SMIC22_04730<br>SMIF22_04930 | 84 | Type II antitoxin YefM | |

[a] *S. cristatus*, *S. equi*, S. *gordonii*, *S. mitis*, *S. oralis*, *S. parasanguinis*, *S. pneumoniae*, *S. pseudopneumoniae*, *S. sanguinis*, and *S. suis* belong to the *Streptococcus* genus.
[b] Criteria: query coverage >80%, amino acid identity >80%, E value <$1e^{-25}$

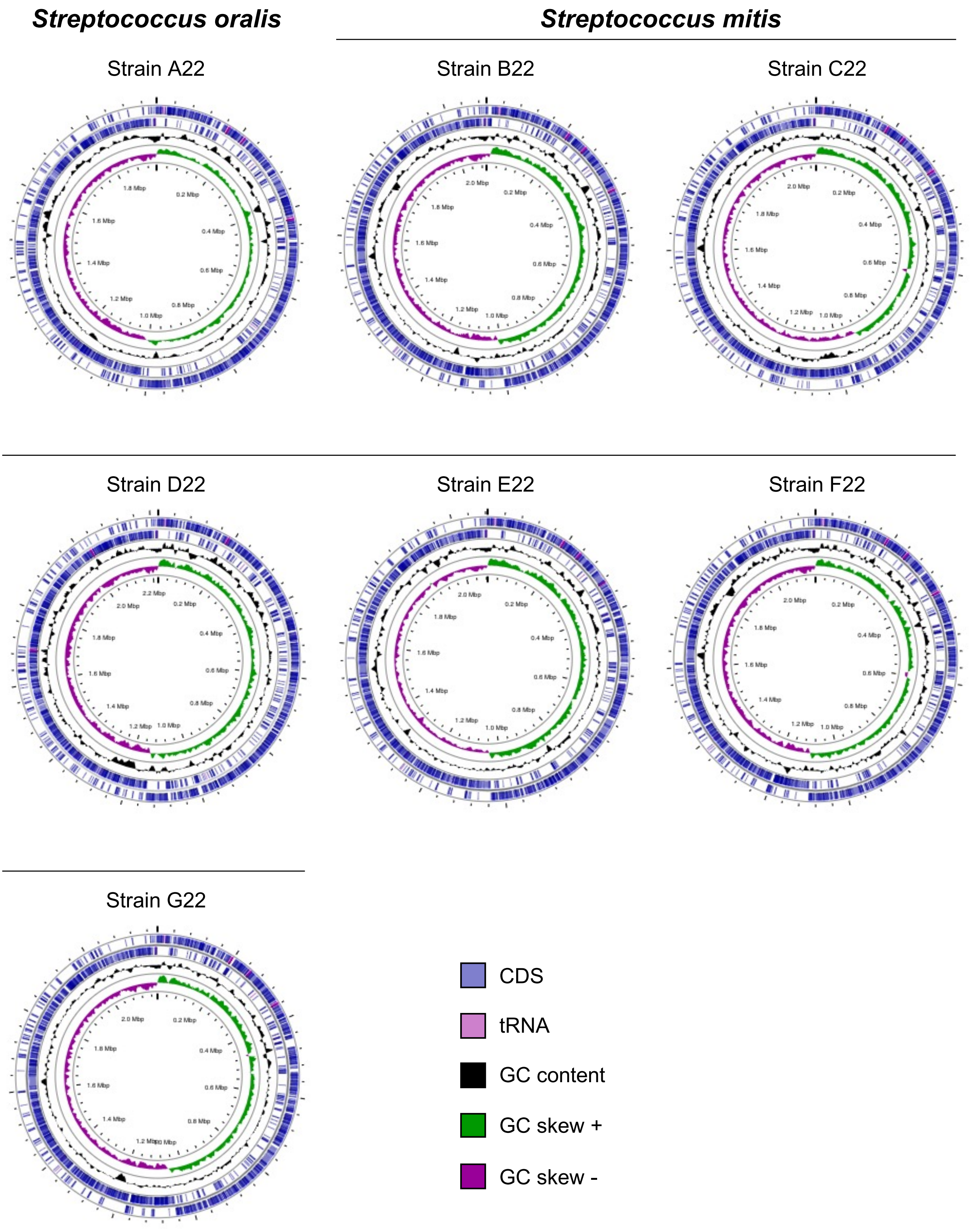


**Figure S1. Circular maps of the genomes of the seven commensal streptococcal study strains.** From the outside to the inside: coding sequences (CDS) identified on the forward and on the reverse strand, respectively, GC content, and GC skew. The circular maps were generated using Proksee.

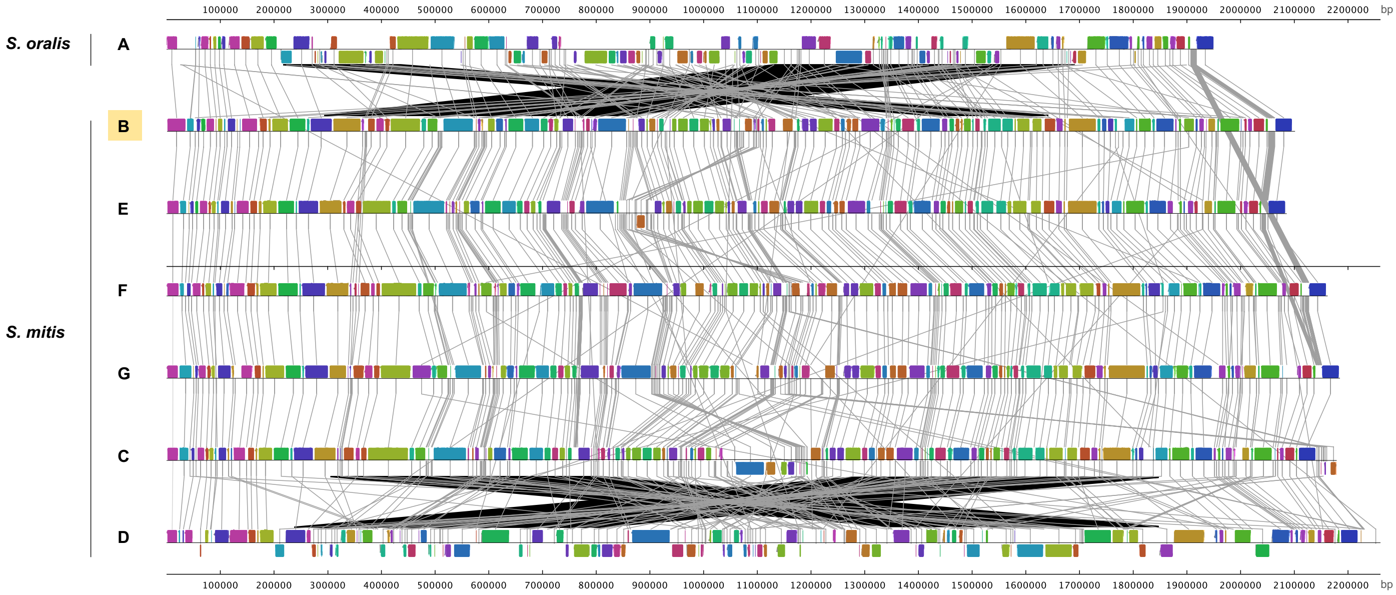


**Figure S2. Genome synteny of the seven commensal streptococcal study strains.** Strain B was set as reference for comparison. Coloured blocks represent homologous regions between, at least, two genomes; if the block is above or below the central line, the region is aligned in the forward or reverse complement orientation, respectively, relative to the reference genome. Blank spaces represent genome-specific regions or presumably homologous regions with internal rearrangements. Grey lines connect homologous regions between genomes. Black regions highlight zones of intense genomic rearrangements. Genome synteny was visualized using Mauve Snapshot 2015-02-25.

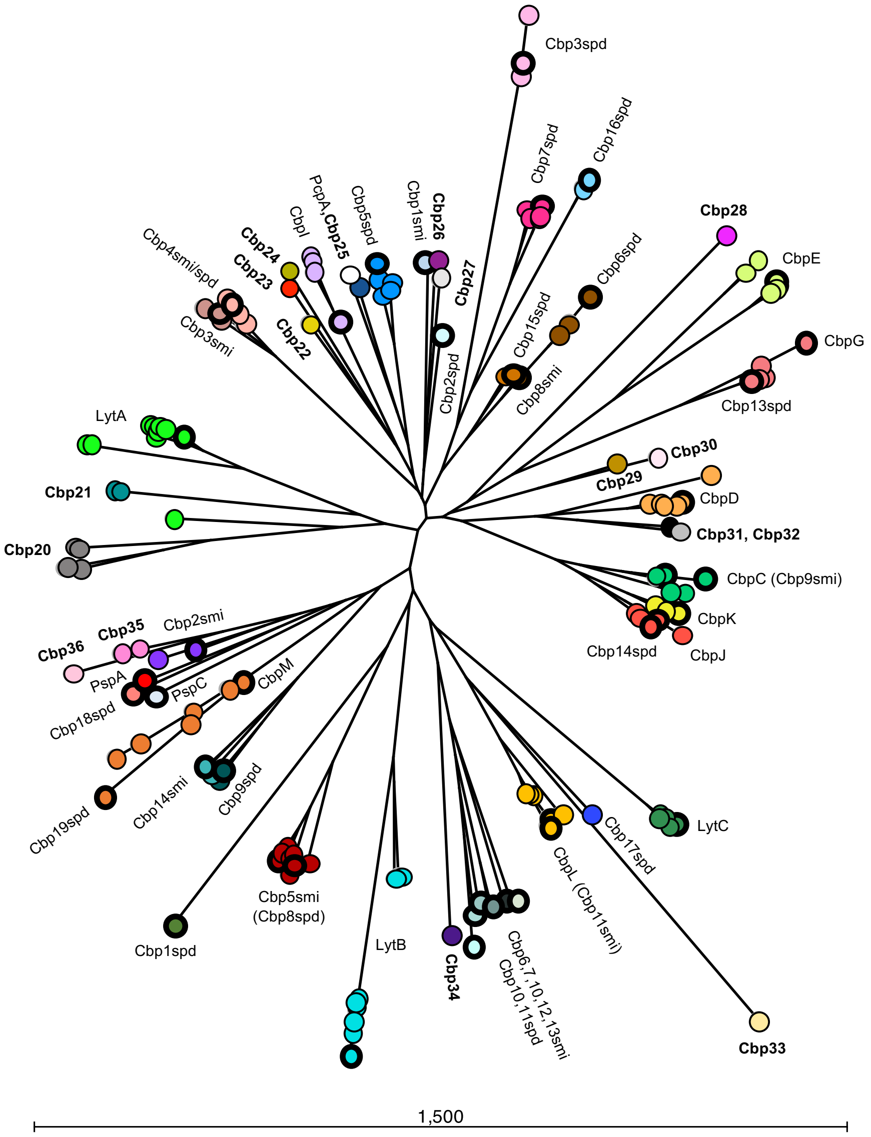


**Figure S3. Phylogenetic analysis of streptococcal choline-binding proteins.** A neighbor-joining tree using the Jukes-Cantor model for protein distance measure and bootstrap analysis based on 500 replicates was generated. The protein sequences (n=178) were retrieved from the seven commensal study strains (131 sequences) and from *S. pneumoniae*, *S. mitis,* and *S. pseudopneumoniae* strains deposited at NCBI database (47 sequences, thicker-line circles). Scale bar reflects the number of amino acid substitutions per sequence site.

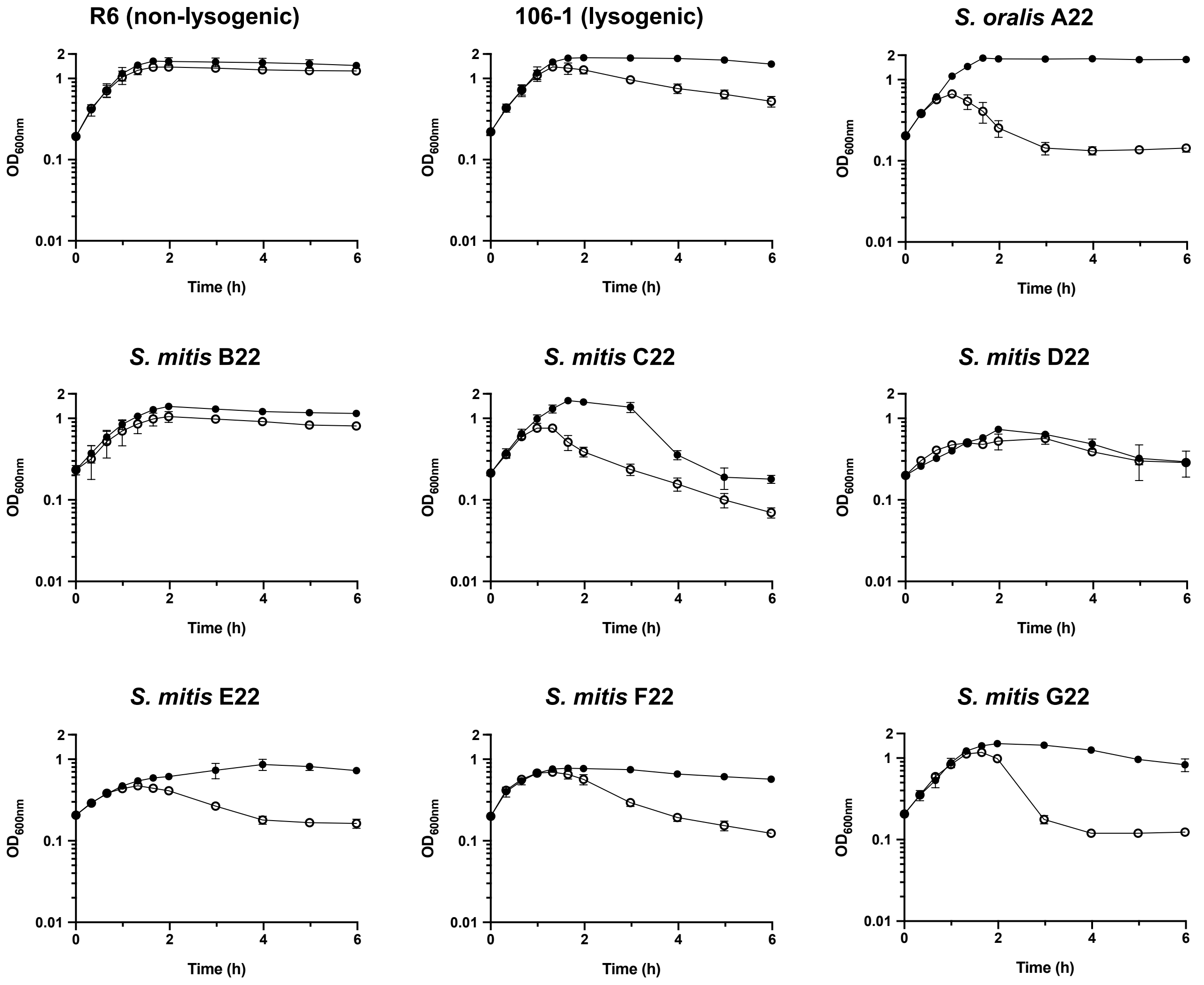


**Figure S4**. **Effect of mitomycin C addition on the growth of the commensal streptococcal study strains.** Cultures grown in THY until an $OD_{600}$ of 0.2 were divided and either left untreated (full circles) or treated with mitomycin C (open circles) at a final concentration of 0.1 µg/mL (strains B22, D22, E22, F22, and G22) or 0.5 µg/ml (strains A22 and C22). Pneumococcal strains R6 and 106-1 were used as controls for strain with no phage (non-lysogenic) and strain with inducible phage (lysogenic), respectively. Experiments were performed three independent times. Error bars represent the mean ± SEM of the three replicates.

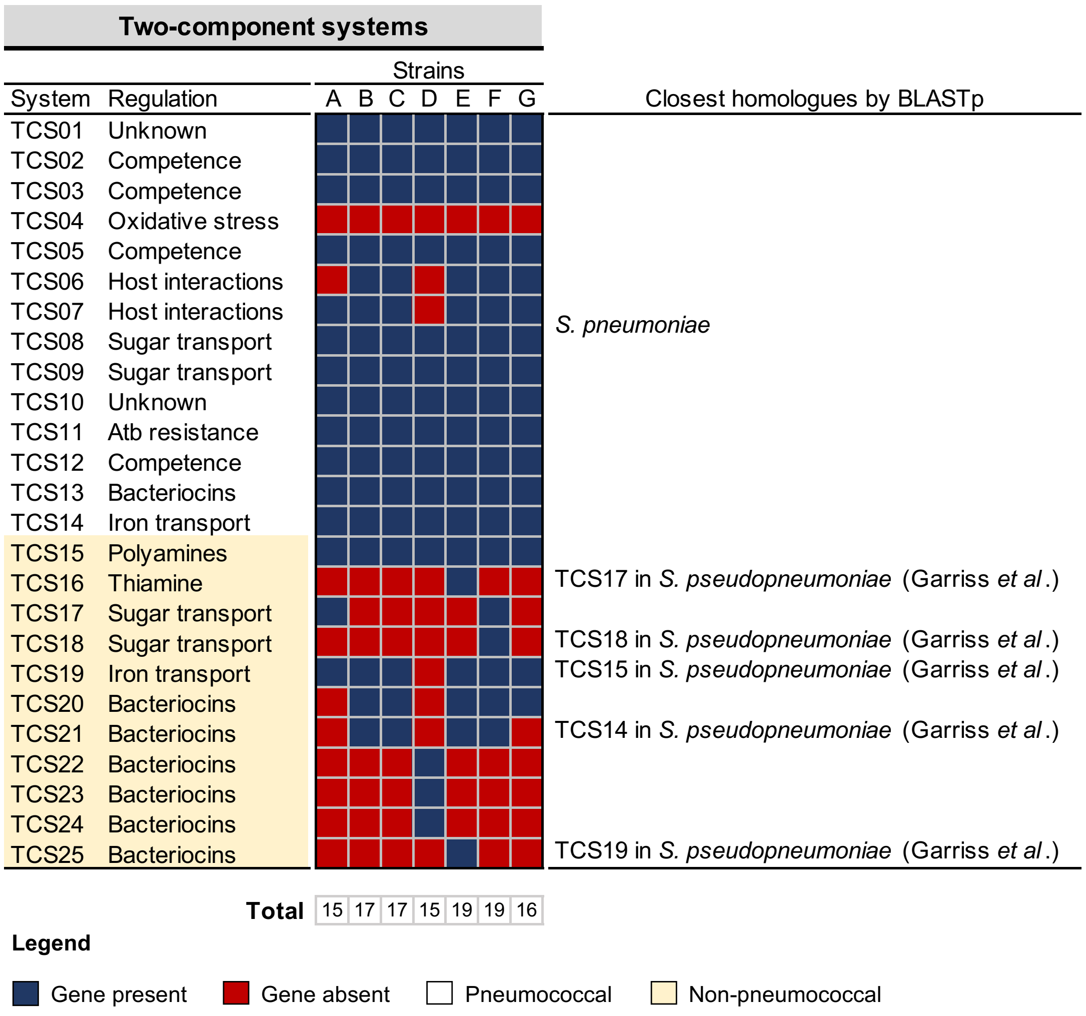


**Figure S5. Distribution of two-component systems (TCS) among the commensal streptococcal study strains.** Blue squares represent TCS genes presence; red squares represent TCS genes absence. Systems in white (TCS01 to TCS14) are homologues to those found in pneumococci, whereas systems highlighted in yellow (TCS15 to TCS25) represent non-pneumococcal TCS.
